# Caspase-3/-7-Specific Metabolic Precursor for Bioorthogonal Tracking of Tumor Apoptosis

**DOI:** 10.1038/s41598-017-16653-2

**Published:** 2017-11-30

**Authors:** Man Kyu Shim, Hong Yeol Yoon, Sangmin Lee, Mun Kyeong Jo, Jooho Park, Jong-Ho Kim, Seo Young Jeong, Ick Chan Kwon, Kwangmeyung Kim

**Affiliations:** 1 Department of Pharmacy, Graduate School, Kyung Hee University, 26, Kyungheedae-ro, Dongdaemun-gu, Seoul, 02447 Republic of Korea; 2 Center for Theragnosis, Biomedical Research Institute, Korea Institute of Science and Technology, 5, Hwarang-ro 14-gil, Seongbuk-gu, Seoul, 02792 Republic of Korea; 3 Department of Pharmacy, College of Pharmacy, Wonkwang University, 460 Iksandae-ro, Iksan, Jeonbuk 54538 Republic of Korea; 4 Department of Life and Nanopharmaceutical Science, Kyung Hee University, 26, Kyungheedae-ro, Dongdaemun-gu, Seoul, 02447 Republic of Korea; 5 KU-KIST Graduate School of Converging Science and Technology, Korea University, 145 Anam-ro, Seongbuk-gu, Seoul, 02841 Republic of Korea

## Abstract

Apoptosis is one of the most important intracellular events in living cell, which is a programmed cell death interrelated with caspase enzyme activity for maintaining homeostasis in multicellular organisms. Therefore, direct apoptosis imaging of living cells can provide enormous advantages for diagnosis, drug discovery, and therapeutic monitoring in various diseases. However, a method of direct apoptosis imaging has not been fully validated, especially for live cells in *in vitro* and *in vivo*. Herein, we developed a new apoptosis imaging technology via a direct visualization of active caspase-3/-7 activity in living cells. For this, we synthesized a caspase-3/-7-specific cleavable peptide (KGDEVD) conjugated triacetylated N-azidoacetyl-D-mannosamine (Apo-S-Ac_3_ManNAz), wherein the Apo-S-Ac_3_ManNAz can be cleaved by the active caspase-3/-7 in live apoptotic cells and the cleaved Ac_3_ManNAz molecules can further generate targetable azido groups (N_3_) on the living cell surface. Importantly, the azido groups on the apoptotic tumor cells could be visualized with Cy5.5-conjugated dibenzylcyclooctyne (DBCO-Cy5.5) via bioorthogonal click chemistry *in vitro* cell culture condition and *in vivo* tumor-bearing mice. Therefore, our Apo-S-Ac_3_ManNAz can be utilized for the further applications in tumor therapy as a monitoring tool for anticancer efficacy and optimization of anticancer new drugs in cell culture system and in tumor-bearing mice.

## Introduction

Over the several decades, molecular imaging based on cellular event monitoring technique is a rapidly emerging in biomedical research for observation of physical, physiological, or metabolic changes in living organisms. It can provide invaluable information of cellular events and molecular pathways as well as mechanisms of various diseases *in vivo* ^1^. Unnatural monosaccharide analogues have provided the way for monitoring metabolic changes via metabolic glycoengineering in pathogen, plants, tumor cells, and living animals^2–5^. Importantly, in combination with bioorthogonal click chemistry, unnatural glycans with various chemical groups, such as ketones, thiols, alkynes, and azides, generated on the cell surface have been used as ‘the artificial chemical receptors’ that irreversibly conjugated to bioorthogonal click chemicals with various imaging agents^4,5^. Therefore, metabolic glycoengineering in combination with bioorthogonal click chemistry can provide visual information of spatiotemporal changes in living subject at cellular or molecular level^6^. In this regard, many researchers have paid much attention to development of enzyme-specific metabolic precursors for targeted cell-specific imaging and drug delivery system^7,8^. In 2010, Bertozzi *et al*. introduced prostate cancer antigen (PSA) protease-specific cleavable peptide conjugated metabolic precursor for prostate tumor tissue-selective imaging^7^. PSA protease-specific metabolic precursor showed the high-specificity to the prostate tumor cells resulting in generating targetable-azido molecules onto the targeted tumor cell surface. We also reported cathepsin B-specific cleavable peptide conjugated metabolic precursor for *in vivo* tumor-specific fluorescence imaging^8^. Our findings showed that targetable azido group generation of cathepsin B-specific metabolic precursor was clearly interrelated with cathepsin B-activity of the tumor cells *in vitro* cell culture system and *in vivo* tumor-bearing mice. Therefore, we expect that the intracellular events in living cells can be able to monitor using target-specific metabolic precursor based on metabolic glycoengineering in combination with bioorthogonal click chemistry.

Among the intracellular events of the cells, apoptosis is a process of programmed cell death including various biochemical events such as cell shrinkage, zeiosis, nuclear fragmentation, and chromatin condensation^9,10^. Highly regulated and controlled biochemical process, apoptosis, is essential to multicellular organisms due to the maintenance of homeostatic balance between proliferation of new cells and death of senescent cells^11^. However, dysregulation of apoptosis lead to various diseases such as Alzheimer’s disease, AIDS, autoimmunity, heart disease, and other disorders including cancer^12–16^. Therefore, observation of apoptosis can provide very valuable information of disorders in living body as well as therapeutic efficacy of drugs during the treatment. In particular, direct visualization and quantification of apoptosis in tumor cells can be utilized for predicting anticancer efficacy and optimizing selection of anticancer drug^17,18^.

For the direct visualization of apoptosis, activity of caspases have been used as a apoptosis-specific target, due to the changes of their activity are interrelated with stages of apoptosis^19^. In particular, caspase-3 (Cas-3) and caspase-7 (Cas-7) are cysteine-aspartic acid proteases which can directly execute of apoptosis followed after sequential activation from activation of caspase-8 (Cas-8) or caspase-9 (Cas-9). Thus, Cas-3/-7-specific cleavable peptide substrate, Asp-Gly-Val-Asp (DEVD), has been extensively used as caspase-cleavable imaging probes for apoptosis imaging for monitoring of caspase activity in tumor cells *in vitro* and *in vivo* conditions^12,20–23^. Other methods to monitor apoptosis *in vitro*, fluorescein isothiocyanate (FITC) conjugated Annexin V (Annexin V-FITC), a protein fluorophore with high affinity to phosphatidylserines during the early stage of apoptosis, and propidium iodide (PI) staining technique, red-fluorescent to detect totally dead cells, are used as a standard protocol for monitoring the progression of apoptosis. Although Annexin V-FITC/PI staining techniques, can discriminate between late apoptotic cells and fully dead cells *in vitro*, they cannot directly monitor Cas-3/-7 activity in living cells and needs additional analysis for Cas-3/-7 activity using western blot analysis and caspase assay kit.

**Figure 1:**
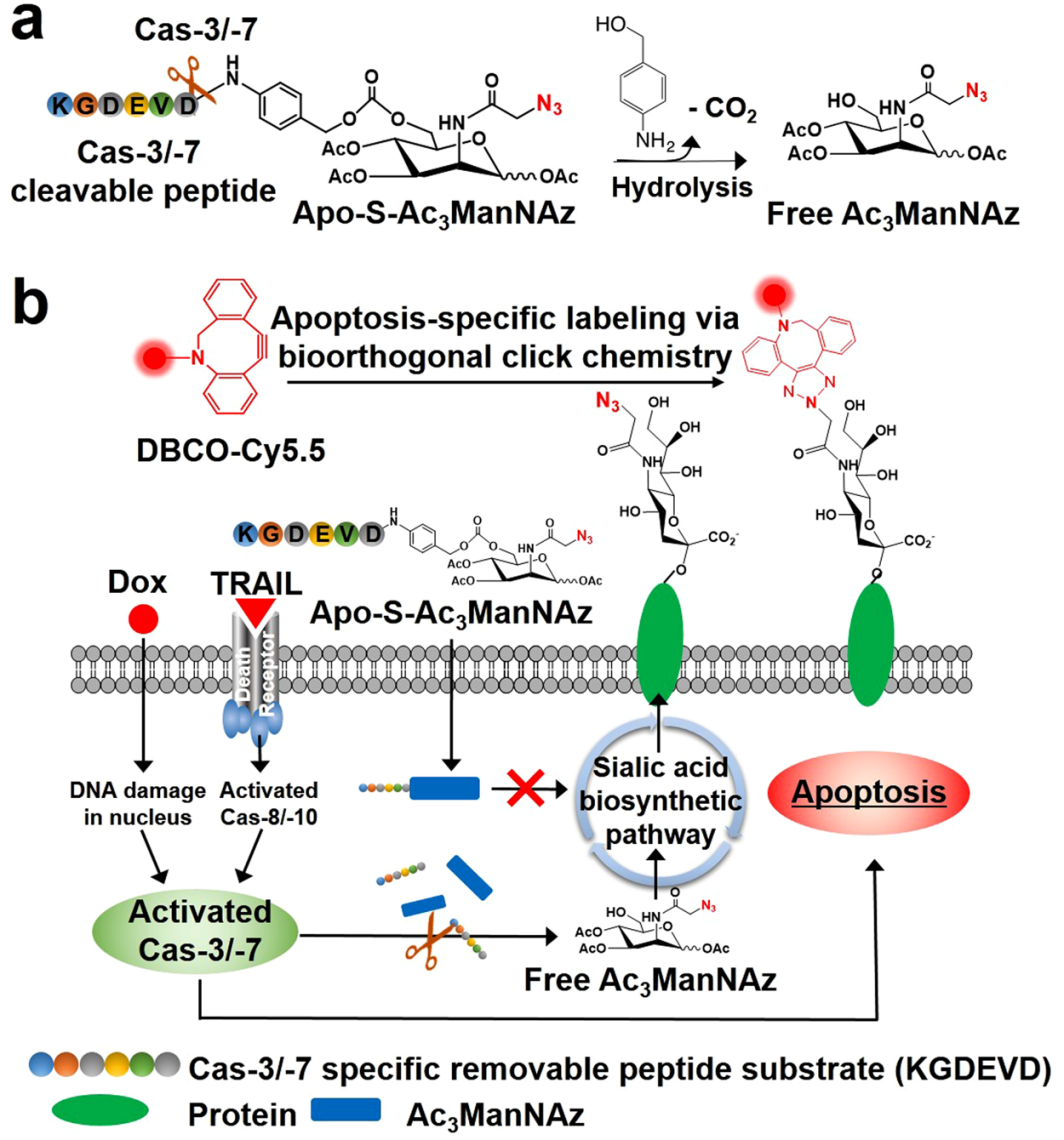
Schematic illustration of Apo-S-Ac_3_ManNAz that can be cleaved to Ac_3_ManNAz by Cas-3/-7 in living cells. (**a**) Cas-3/-7-specific cleavage of Apo-S-Ac_3_ManNAz to a linker (S), KGDEVD peptide, and Ac_3_ManNAz. (**b**) Apoptosis-specific molecular imaging between exogenous azido groups and DBCO-Cy5.5 via bioorthogonal click chemistry in living cells.

To overcome these limitations for apoptosis-specific molecular imaging, we developed a Cas-3/-7-specific cleavable peptide conjugated unnatural monosaccharide as a new metabolic precursor that can be specifically cleaved by the active Cas-3/-7 in the cytoplasm of the tumor cells. This is because the Cas-3/-7 has been known to be a cysteine protease, which is major executioner caspase during the early stage of apoptosis in living cells. As a new apoptosis imaging probe, Cas-3/-7-specific cleavable peptide substrate (Lys-Gly-Asp-Glu-Val-Asp, KGDEVD) was directly conjugated to metabolic precursor that is composed of three major compartments, Cas-3/-7-specific cleavable peptide substrate (KGDEVD), self-immolative linker of *p*-aminobenzyloxycarbonyl (PABC) (S), and the unnatural monosaccharide of triacetylated N-azidoacetyl-D-mannosamine (Ac_3_ManNAz), respectively, resulting in Apo-S-Ac_3_ManNAz (Fig. 1a). We already confirmed that the Cas-3/-7-specific cleavable peptide substrate of KGDEVD was successfully cleaved by the Cas-3/-7 through *in vitro* enzyme reaction and *in vivo* tumor-bearing mice^20,21,24^. After cellular uptake of Apo-S-Ac_3_ManNAz, importantly, the KGDEVD peptide substrate can be selectively cleaved from Apo-S-Ac_3_ManNAz by Cas-3/-7 which can be activated during the apoptosis triggered by anticancer drugs (intrinsic pathway) or tumor necrosis factor-related apoptosis-inducing ligand (TRAIL) (extrinsic pathway) (Fig. 1b **)**. In addition, cleaved S-Ac_3_ManNAz can be finally hydrolyzed to give free Ac_3_ManNAz which can be used for generating azido (-N_3_) groups on the tumor cell surface through sialic acid biosynthetic pathway. Finally, apoptosis can be visualized with a near infrared fluorescence (NIRF) dye conjugated dibenzylcyclooctyne (DBCO-Cy5.5) via bioorthogonal click chemistry *in vitro* and *in vivo*. Therefore, we expect that the Cas-3/-7-specific Apo-S-Ac_3_ManNAz can generate azido groups on the apoptotic cell surface and they can be used for the direct apoptosis visualization at the early stage of apoptosis via bioorthogonal click chemistry in living cells *in vitro* and *in vivo*.

To obtain Cas-3/-7-specific cleavable metabolic precursor, Cas-3/-7-specific cleavable peptide substrate of KGDEVD was conjugated to Ac_3_ManNAz with a self-immolative linker of PABC, resulting in KGDEVD-PABC-Ac_3_ManNAz (Apo-S-Ac_3_ManNAz) (Figure S1, compound 5). It has been known that the Cas-3/7 can cleave specifically the N-terminus of tetra motif of DEVD^25^. Purification of Apo-S-Ac_3_ManNAz was performed using reverse phase-high performance liquid chromatography (RP-HPLC), wherein the purity of Apo-S-Ac_3_ManNAz was about 99%. The chemical structure of Apo-S-Ac_3_ManNAz was analyzed based on the representative characteristic peaks from ^1^H-NMR analysis, where 1.7–2.0 ppm (-CH_3_ at Ac_3_ManNAz), 7.30 and 7.66 ppm (-CH at PABC linker) (Figure S2a and S2b). Finally, the molecular weight of Apo-S-Ac_3_ManNAz was measured using electrospray ionization mass spectrometry (ESI-MS, m/z calculated: 1222.46, found: 1223.44 [M+H^+^]) and matrix-assisted laser desorption/ionization analysis (MALDI, m/z calculated: 1222.46, found 1223.44, 1245.45 [M+Na^+^] and 1267.40 [M+2Na^+^]), respectively (Figure S3).

**Figure 2:**
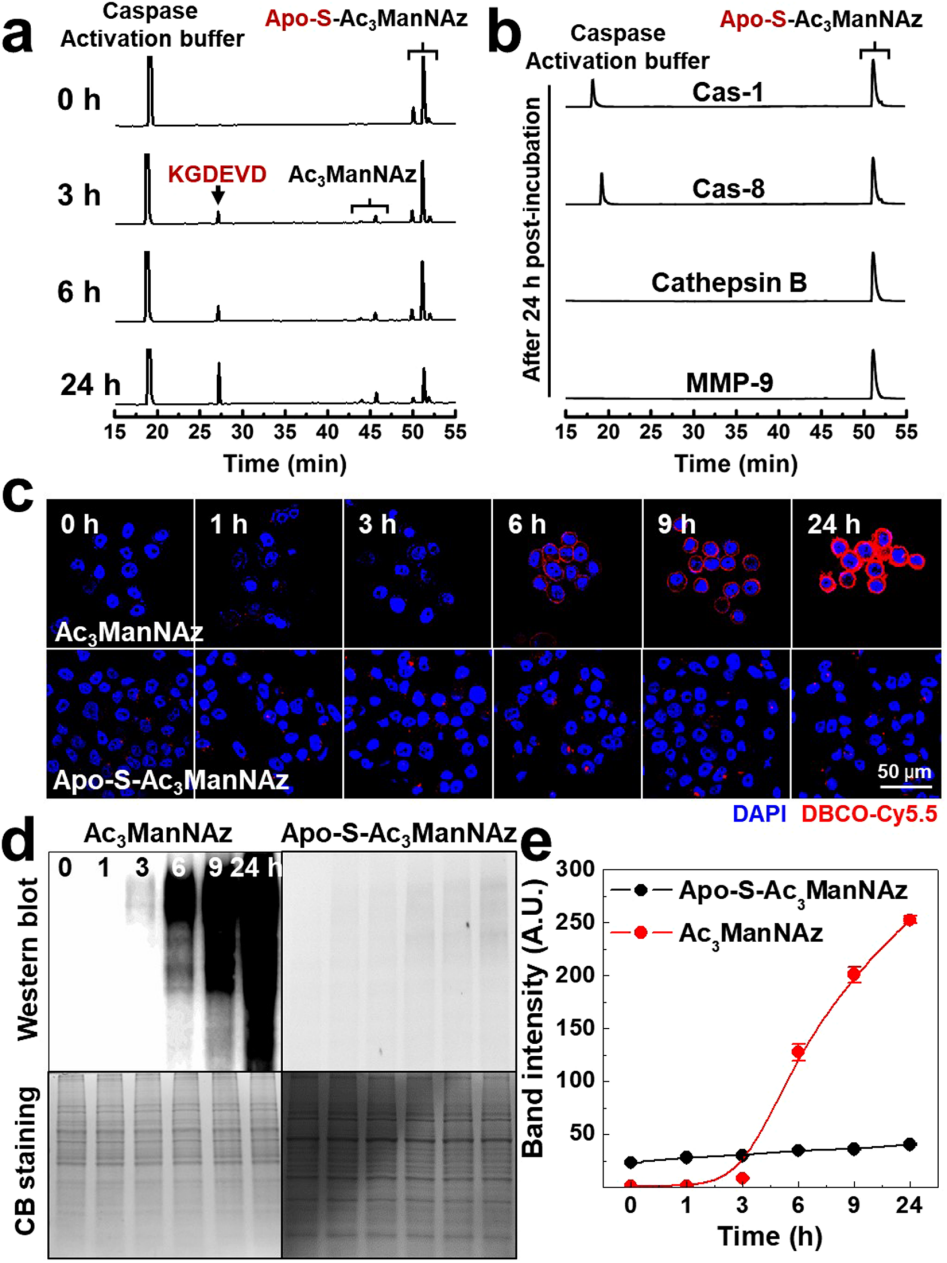
(**a**) *In vitro* cleavage of Apo-S-Ac_3_ManNAz was monitored using HPLC system at 0, 3, 6, and 24 h post-incubation with Cas-3 (15 μg/ml). (**b**) As a control experiment, Apo-S-Ac_3_ManNAz was also monitored using HPLC system at 24 h post-incubation with 15 μg/ml of Cas-1, Cas-8, Cathepsin B and MMP-9, respectively. The generation of azido groups on the surface of non-apoptotic PC-3 tumor cells *in vitro*. (**c**) Time-dependent CLSM images of Ac_3_ManNAz (20 μM) or Apo-S-Ac_3_ManNAz (20 μM)-treated PC-3 tumor cells, followed by DBCO-Cy5.5 (200 nM) to visualize azido groups on the cell surface. Red = DBCO-Cy5.5 channel; Blue = DAPI channel. (**d**) Western blot analysis of azido groups (N_3_) containing glycoproteins in cell lysates. The coomassie blue (CB) stain shows the total amount of glycoproteins in cell lysates. (**e**) Quantification of band intensity of azido groups of Western blot data using ImageJ software.

The Cas-3-specific degradation of Apo-S-Ac_3_ManNAz was clearly observed in the presence of Cas-3 (15 μg/ml) by HPLC analysis (Fig. 2a). After 3 h post-incubation with the active Cas-3, the cleaved KGDEVD peptide from Apo-S-Ac_3_ManNAz was clearly observed and the amount of cleaved KGDEVD peptide consistently increased up to 24 h, indicating the rapid and successive Cas-3-specfiic cleavage of KGDEVD substrate from Apo-S-Ac_3_ManNAz. Moreover, the amount of cleaved Ac_3_ManNAz from Apo-S-Ac_3_ManNAz gradually increased in a time-dependent manner. The enzyme cleavage reaction between Apo-S-Ac_3_ManNAz and Cas-7 was also confirmed by HPLC analysis after 24 h post-incubation of Cas-7. As expected, the characteristic peaks of KGDEVD and Ac_3_ManNAz were clearly observed at 24 h post-incubation, indicating Ac_3_ManNAz could be cleaved from Apo-S-Ac_3_ManNAz in the presence of Cas-7 (Figure S4a). As control, in the absence of Cas-3 and Cas-7, however, observable changes of Apo-S-Ac_3_ManNAz could not be monitored in HPLC analysis (Figure S4b). This is because the structure of Apo-S-Ac_3_ManNAz is very stable under physiological condition. Furthermore, when Apo-S-Ac_3_ManNAz was incubated with Cas-1, Cas-8, cathepsin B, and matrix metalloproteinase 9 (MMP-9), respectively, we could not observe any cleavage of Ac_3_ManNAz from Apo-S-Ac_3_ManNAz up to 24 h (Fig. 2b). These enzyme reactions of Apo-S-Ac_3_ManNAz with Cas-3, Cas-7, Cas-1, Cas-8, cathepsin B, and MMP-9 clearly showed that enzyme-specific cleavage of Apo-S-Ac_3_ManNAz was only observed in the presence of Cas-3 and Cas-7. Although previous report showed that DEVD can be cleaved by cathepsin B, legumain, Cas-8, and others, we could not observed any peaks of released Ac_3_ManNAz from Apo-S-Ac_3_ManNAz when it incubated with cathepsin B or Cas-8. Thus, we thought that KGDEVD has more specificity against Cas-3 and Cas-7 not cathepsin B or Cas-8 than DEVD^26,27^.

Next, we confirmed the generation of azido groups into the non-apoptotic tumor cells via metabolic glycoengineering using confocal laser scanning microscopy (CLSM). PC-3 human prostate cancer cells were pre-incubated with Apo-S-Ac_3_ManNAz (20 μM) or Ac_3_ManNAz (20 μM) for 0, 1, 3, 6, 9, and 24 h, respectively, and then the PC-3 tumor cells were treated with DBCO-Cy5.5 (200 nM) for 2 h to visualization of azido groups (Fig. 2c). As expected, the CLSM images of Ac_3_ManNAz-treated PC-3 tumor cells showed that NIRF intensity gradually increased in a time-dependent manner up to 24 h. However, there was no observable changes when the PC-3 tumor cells were treated with Apo-S-Ac_3_ManNAz. This is because Apo-S-Ac_3_ManNAz was very stable without active Cas-3/-7 in healthy PC-3 tumor cells. In addition, the PC-3 tumor cells showed only negligible morphological changes during the treatment of Ac_3_ManNAz or Apo-S-Ac_3_ManNAz (Figure S5). The generation of azido groups in glycoproteins were further analyzed using western blot analysis (Fig. 2d). The amount of azido groups containing sialic acid in lysates of PC-3 tumor cells were chemically labeled with phosphine-PEG_3_-biotin via formation of triazole linkage for 6 h and then separated by molecular weight using 10% sodiumdodecylsulfate (SDS)-polyacrylamide gel electrophoresis. The separated azido groups contained sialic acid were transferred onto PVDF membranes, followed by visualization using streptavidin-HRP. As shown in Fig. 2d, the band intensity of azido group contained sialic acid gradually increased in a time-dependent manner for 24 h when PC-3 tumor cells were treated with Ac_3_ManNAz. However, compare to the Ac_3_ManNAz treated PC-3 tumor cells, there was no observable changes when they were treated with Apo-S-Ac_3_ManNAz (Fig. 2e). This is because the modification of C6 position at Ac_3_ManNAz using the protection molecules, such as cathepsin-B cleavable peptide of KGRR and prostate cancer antigen (PSA) protease-specific cleavable peptide of HSSKLY could interfere with the phosphorylation of C6-OH via ManNAc-6-kinase, a master regulator of sialic acid synthesis, during the sialic acid synthesis in the tumor cells^7,8,28,29^. These results confirmed that the Apo-S-Ac_3_ManNAz cannot present any apoptotic signal of the tumor cells without any apoptosis triggers.

**Figure 3:**
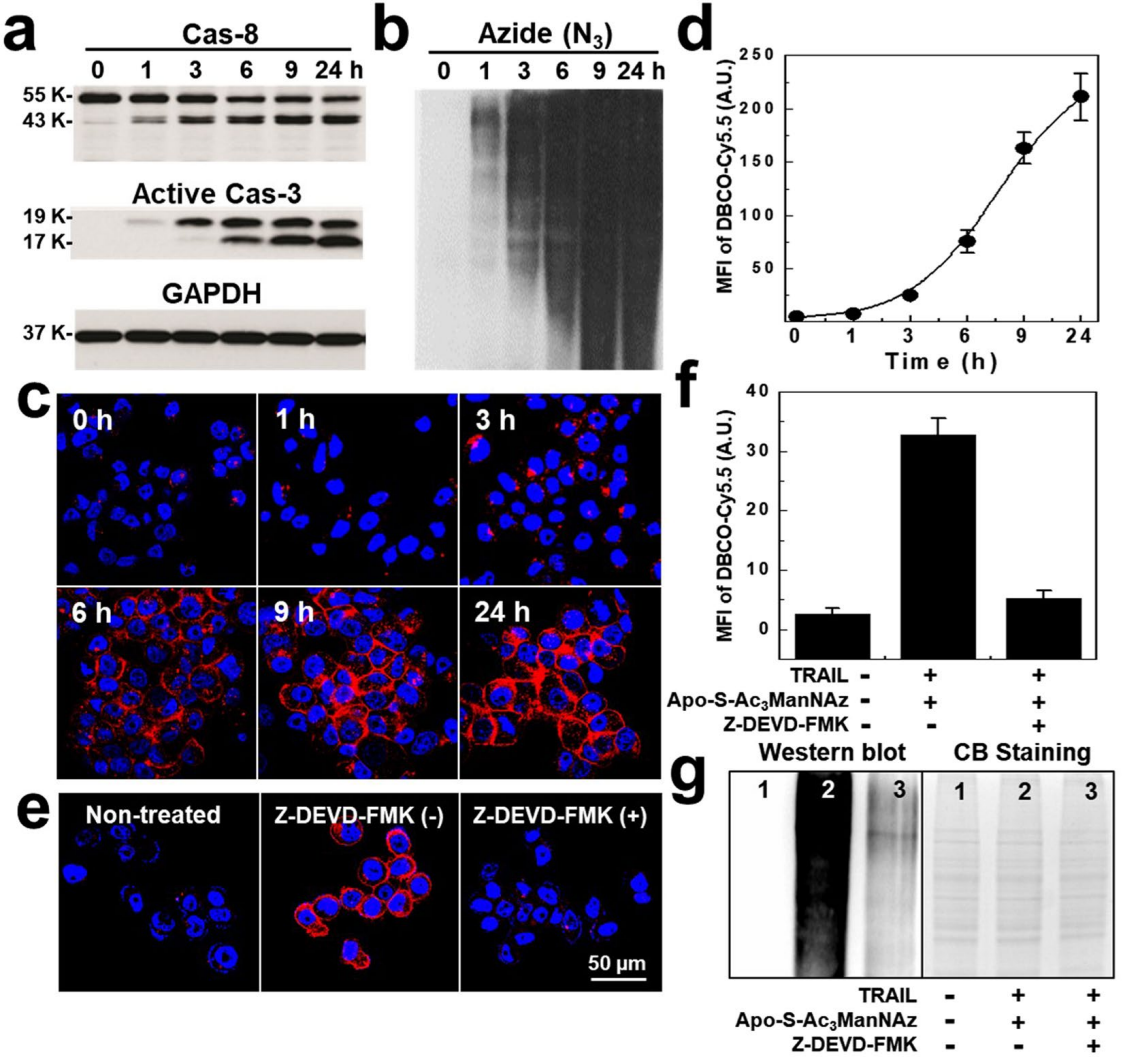
*In vitro* generation of apoptosis-specific azido groups of Apo-S-Ac_3_ManNAz-treated PC-3 tumor cells. (**a**) Time-dependent TRAIL-induced apoptosis was analyzed using western blot analysis of Cas-3, Cas-8 and GAPDH, wherein PC-3 tumor cells were incubated with TRAIL for 0, 1, 3, 6, 9, and 24 h for inducing apoptosis. (**b**) Time-dependent generation of azido groups was analyzed by western blot analysis of Apo-S-Ac_3_ManNAz- and TRAIL-treated PC-3 tumor cells. (**c**) Time-dependent CLSM images of Apo-S-Ac_3_ManNAz (20 μM) and TRAIL (7 ng/ml)-treated PC-3 tumor cells, followed by DBCO-Cy5.5 (200 nM) to visualize azido groups. Red = DBCO-Cy5.5 channel; Blue = DAPI channel. (**d**) The MFI of Apo-S-Ac_3_ManNAz- and TRAIL-treated PC-3 tumor cells at various incubation time. (**e**) CLSM images of apoptosis-specific Apo-S-Ac_3_ManNAz-treated PC-3 tumor cells after post-treatment of TRAIL or TRAIL with z-DEVD-FMK at 24 h. Red = DBCO-Cy5.5 channel; Blue = DAPI channel. (**f**) MFI of flow cytometry analysis of Apo-S-Ac_3_ManNAz- and TRAIL-treated PC-3 tumor cells without/with z-DEVD-FMK at 24 h. (**g**) Western blot analysis of generation of azido groups of Apo-S-Ac_3_ManNAz-treated PC-3 tumor cells after post-treatment of TRAIL or TRAIL with z-DEVD-FMK at 24 h.

To confirm the generation of azido groups on the surface of apoptotic tumor cells, we firstly tested the cellular toxicity of Apo-S-Ac_3_ManNAz-treated tumor cells *in vitro*. As expected, Apo-S-Ac_3_ManNAz-treated PC-3 tumor cells showed any significant cytotoxicity, wherein in the concentration of Apo-S-Ac_3_ManNAz increased from 0 to 100 μM (Figure S6a). In addition, the proliferation of PC-3 tumor cells was not inhibited after incubating with 20 μM of Apo-S-Ac_3_ManNAz for 3 days, indicating the non-toxicity of Apo-S-Ac_3_ManNAz in the cell culture system (Figure S6b). For the Cas-3-specific apoptosis imaging, we choose the tumor necrosis factor-related apoptosis-inducing ligand (TRAIL) as a model apoptosis trigger, which can selectively triggered apoptosis in the various tumor cells^30^. The cell viability of PC-3 tumor cells showed concentration-dependently decreased up to 20% when they were treated with 5 to 200 ng/ml of TRAIL for 24 h (Figure S6c). In addition, the morphological changes of PC-3 tumor cells, such as shrinking and rounding, were clearly observed at 9 h and 24 h post-incubation of TRAIL (7 ng/ml) (Figure S6d). As shown in the Figure S7, cell viability of PC-3 tumor cells pre-treated with 20 μM of Apo-S-Ac_3_ManNAz was higher than that of non-treated PC-3 tumor cells after 7 ng/ml of TRAIL treatment. This is because that exogenous Apo-S-Ac_3_ManNAz which containing Cas-3/-7 substrate (DEVD) could compete with endogenous substrates in the PC-3 tumor cells. However, cell viability of PC-3 tumor cells pre-treated with 20 μM of Apo-S-Ac_3_ManNAz was also gradually decreased in a TRAIL dose-dependent manner, indicating that apoptosis could be successfully occurred in PC-3 tumor cells. In particular, when the PC-3 tumor cells were treated with 7 ng/ml of TRAIL, the band intensity on the western blot data of activated Cas-3 and Cas-8 in the lysates of PC-3 tumor cells increased according to the incubation time (Fig. 3a). This result supports that TRAIL could trigger apoptosis of PC-3 tumor cells via activating of Cas-3 and Cas-8 that are the hallmark caspase cascade characteristic of the apoptotic pathway. The band intensity of the azido groups in glycoproteins of PC-3 tumor cells increased with incubation time after treatment of 7 ng/ml of TRAIL, indicating successfully generation of azido groups on the surface of PC-3 tumor cells (Fig. 3b). These results support that the generation of azido groups on the apoptotic PC-3 tumor cells was strongly interrelated with the increase amount of active Cas-3 and Cas-8 after triggering apoptosis using TRAIL. Under optimal condition, to introduce of azido groups in apoptotic PC-3 tumor cells, the PC-3 tumor cells were pre-incubated with 20 μM of Apo-S-Ac_3_ManNAz for 4 h before treatment of TRAIL. And then 7 ng/ml of TRAIL was treated for 0, 1, 3, 6, 9, and 24 h, respectively. To visualization of azido groups in apoptotic PC-3 tumor cells, the PC-3 tumor cells were treated with DBCO-Cy5.5 (200 nM) for 2 h. The high resolution CLSM images of Apo-S-Ac_3_ManNAz and TRAIL-treated PC-3 tumor cells showed the morphological shrinkage, due to the induced apoptosis. And, the NIRF intensity also gradually increased according to the incubation time of TRAIL (Fig. 3c and Figure S8a). The mean fluorescence intensities (MFI) of PC-3 tumor cells treated with Apo-S-Ac_3_ManNAz and TRAIL gradually increased 1.6, 5.3, 15.8, 34.2, and 44.3 fold higher than only Apo-S-Ac_3_ManNAz-treated PC-3 tumor cells at 1, 3, 6, 9, and 24 h, respectively (Fig. 3d). To observe Cas-3-specific generation of azido (N_3_) groups by Apo-S-Ac_3_ManNAz, we additionally estimated bioorthogonal apoptosis tracking efficacy of Apo-S-Ac_3_ManNAz using both LNCaP (human prostate carcinoma) cell line and HT-29 (human colorectal adenocarcinoma) cell line. LNCap tumor cells showed 21-folds higher DBCO-Cy5.5 fluorescence when it was induced apoptosis by TRAIL compared to the Apo-S-Ac_3_ManNAz-treated LNCaP tumor cells (Figure S9a and S9b). Furthermore, HT-29 tumor cells showed 11-folds higher DBCO-Cy5.5 fluorescence when it was induced apoptosis by TRAIL compared to the Apo-S-Ac_3_ManNAz-treated HT-29 tumor cells (Figure S9c and S9d). This is because that Apo-S-Ac_3_ManNAz can release Ac_3_ManNAz which can generate targetable azido groups onto the cell surface by Cas-3/-7-specific cleavage reaction, regardless of cell types. Therefore, we expect that Apo-S-Ac_3_ManNAz can be utilized for monitoring apoptosis in various tumor cells.

As a control experiment, we carefully tested *in vitro* Cas-3/-7 specificity of Apo-S-Ac_3_ManNAz using Cas-3/-7 inhibitor, N-benzyloxycarbonyl-Asp(OMe)-Glu(OMe)-Val-Asp(OMe)-fluoromethylketone (z-DEVD-FMK)^31^. Morphological changes of PC-3 tumor cells were observed when they were treated with Apo-S-Ac_3_ManNAz (20 μM) and TRAIL (7 ng/ml) for 24 h. Importantly, Apo-S-Ac_3_ManNAz, TRAIL and z-DEVD-FMK (200 μM**)** treated PC-3 tumor cells showed negligible morphological changes, indicating apoptosis was successfully inhibited by z-DEVD-FMK (Figure S8b). Furthermore, z-DEVD-FMK-treated PC-3 tumor cells showed only negligible changes of NIRF signal of DBCO-Cy5.5-treated PC-3 tumor cells (Fig. 3e). The MFI of Apo-S-Ac_3_ManNAz and TRAIL- treated PC-3 tumor cells was 6.29-fold higher than that of z-DEVD-FMK-treated cells (Fig. 3f). Finally, the azido groups on the z-DEVD-FMK-treated cells were greatly suppressed as much as non-treated cells, indicating the higher specificity of Apo-S-Ac_3_ManNAz against Cas-3/-7 activity in apoptotic PC-3 tumor cells (Fig. 3g). Additionally, we confirmed the inhibition of azido group generation by other enzyme inhibitor, z-FA-FMK. z-FA-FMK is inhibitor of cathepsin B which can partially prevent p53-dependent apoptosis and inhibit certain caspase, such as Cas-2, -3, -6, and -7^32–34^. As shown in Figure S10a, the strong fluorescence intensity of DBCO-Cy5.5 was observed in both TRAIL- and TRAIL/z-FA-FMK-treated PC-3 tumor cells, indicating that azido groups were successfully generated onto the cell surfaces. Notably, the fluorescence intensity in z-FA-FMK-treated PC-3 tumor cells was 26.5% reduced, compared to the Apo-S-Ac_3_ManNAz/TRAIL-treated PC-3 tumor cells (Figure S10b). However, the fluorescence intensity in z-FA-FMK-treated PC-3 tumor cells was enough to discriminate apoptotic cells, which was 13.5-folds higher than non-apoptotic cells.

**Figure 4:**
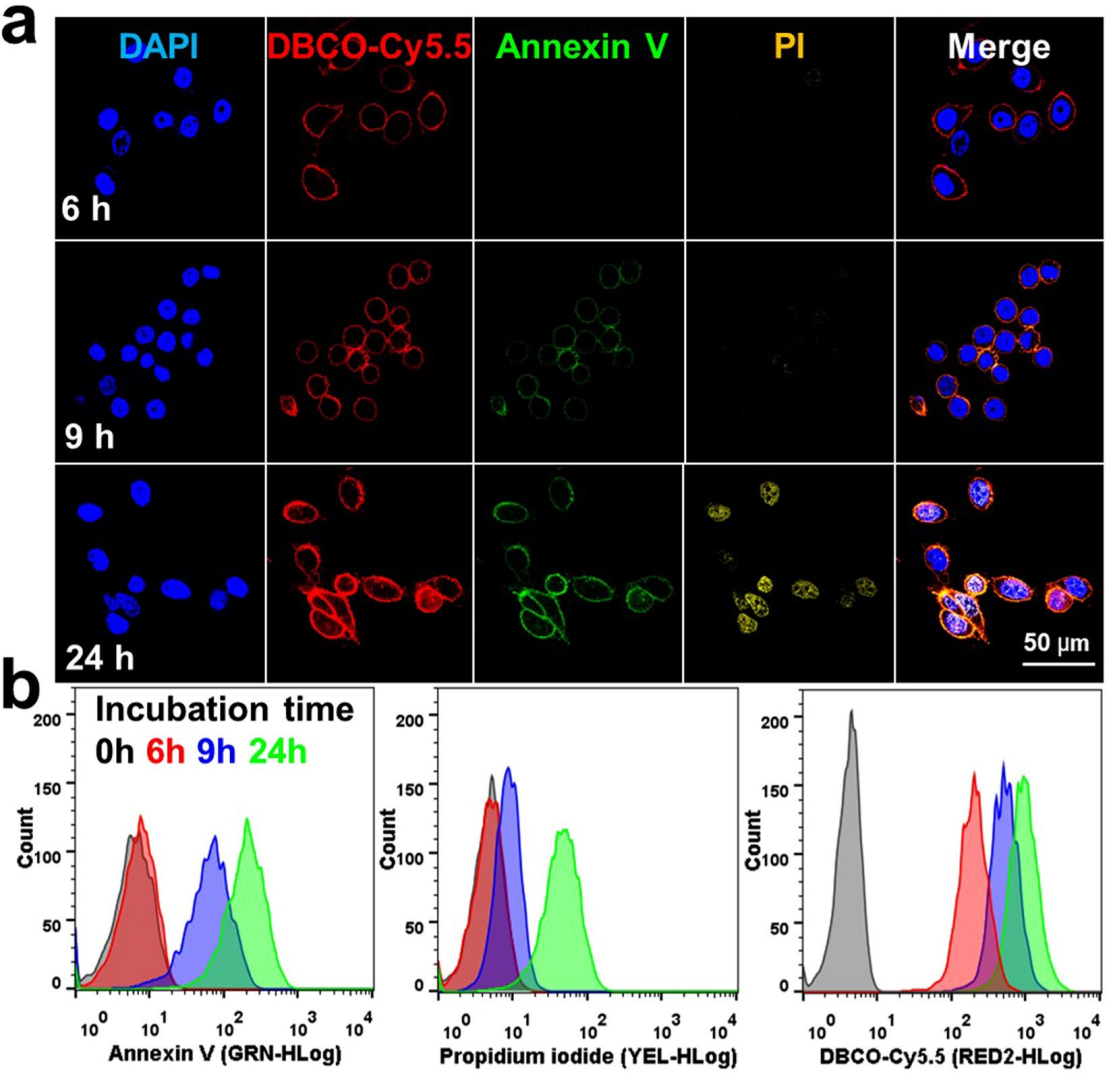
CLSM imaging of apoptosis-specific PC-3 tumor cells. (**a**) PC-3 tumor cells were incubated with the Apo-S-Ac_3_ManNAz (20 μM) for 4 h and further treated with TRAIL (7 ng). The generation of Cas-3-specific azido groups were visualized with DBCO-Cy5.5 after 2 h post-incubation of TRAIL. Annexin V-FITC and PI staining for imaging of apoptotic PC-3 tumor cells. Red = DBCO-Cy5.5 channel; Blue = DAPI channel; Green = Annexin V-FITC channel; Yellow = PI channel. (**b**) FACS analysis data of Apo-S-Ac_3_ManNAz- and TRAIL-treated PC-3 tumor cells, wherein the cells were labeled with DBCO-Cy5.5, Annexin V-FITC and PI staining after 6 h, 9 h and 24 h post-incubation with TRAIL, respectively.

Next, we observed apoptosis sensitivity of Apo-S-Ac_3_ManNAz-treated PC-3 tumor cells using CLSM and flow cytometry (FACS) analysis *in vitro*. As control, Annexin V-FITC and PI staining were performed together as a gold standard to identify the characteristic of cell death after treatment of TRAIL. First, the PC-3 tumor cells were incubated with Apo-S-Ac_3_ManNAz for 4 h before TRAIL treatment and then 7 ng/ml of TRAIL was added to the PC-3 tumor cells to induce apoptosis. Figure 4a showed that the fluorescence signal of DBCO-Cy5.5-treated PC-3 tumor cells gradually increased with the incubation time of TRAIL at 6, 9, and 24 h. This is because Apo-S-Ac_3_ManNAz was successfully cleaved into Ac_3_ManNAz by activated Cas-3, and leads to generate targetable azido groups that irreversibly conjugated to bioorthogonal click group of DBCO-Cy5.5 on the PC-3 tumor cell surface. However, the fluorescence of Annexin V-FITC was only observed after 9 h post-treatment of Trail and PI stain was lately observed at 24 h post-treatment of TRAIL. Importantly, the PC-3 tumor cells did not present any fluorescence signals of both Annexin V-FITCand PI at 6 h post-treatment of TRAIL, indicating that Apo-S-Ac_3_ManNAz can visualize the early stage of apoptosis, compared to that of Annexin V-FITC- and PI-treated PC-3 tumor cells. Furthermore, FACS analysis data showed that Apo-S-Ac_3_ManNAz-treated PC-3 tumor cells presented the high fluorescence intensity only after 6 h post-treatment of TRAIL and the fluorescence intensity gradually increased up to 24 h (Fig. 4b). However, the fluorescence intensity of Annexin V-FITC-or PI-treated PC-3 tumor cells was observed at 9 h or 24 h post-treatment of TRAIL, indicating later apoptosis detection of PC-3 tumor cells than Apo-S-Ac_3_ManNAz^35^. This is because Annexin V-FITC can bind to phosphatidylserines during the late stage of apoptosis and PI staining only detects the totally dead cells, compared to the Cas-3/-7-related early stage of apoptosis *in vitro* imaging data. Based on these cellular imaging data, we confirmed that Apo-S-Ac_3_ManNAz has potential for the Cas-3 activity-related direct apoptosis imaging in early-stage of apoptotic cells.

**Figure 5:**
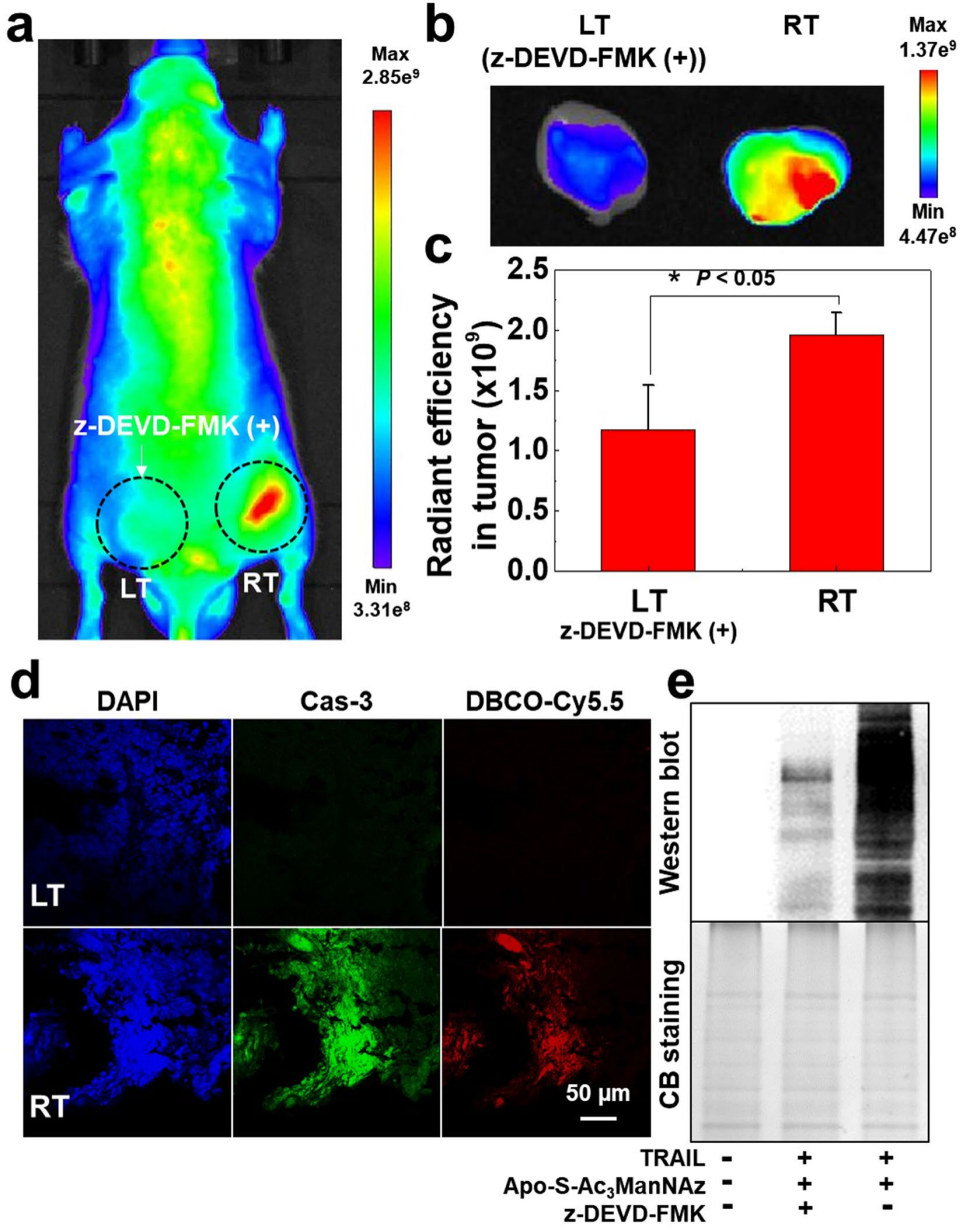
Non-invasive apoptosis imaging of Apo-S-Ac_3_ManNAz- and TRAIL-treated tumor tissues in PC-3 tumor-bearing mice model (n = 5). For Cas-3-specific generation of azido groups in PC-3 tumor tissues, Apo-S-Ac_3_ManNAz (3 mg/kg) was directly injected both of left ((+) z-DEVD-FMK) and right ((−) z-DEVD-FMK) tumor tissues 4 times. (**a**) NIRF images of azido groups in PC-3 tumor tissues labeled with DBCO-Cy5.5. (**b**) *Ex vivo* fluorescence images of left ((+) z-DEVD-FMK) and right ((−) z-DEVD-FMK) tumor tissues. (**c**) NIRF intensity of PC-3 tumor tissues (n = 5) was quantified by Living Image software. (*) indicates difference at the *p* < 0.05 significance level. (**d**) NIRF images of PC-3 tumor tissues from (b). Cas-3 in PC-3 tumor tissues was stained by immunofluorescence (IF) stain with FITC-conjugated active Cas-3 antibody. Red = DBCO-Cy5.5 channel; Green = Active Cas-3 antibody channel; Blue = DAPI channel. (**e**) Western blot analysis of azido groups-containing glycoproteins *in vivo* PC-3 tumor tissues.

In order to demonstrate the feasibility of non-invasive apoptosis imaging *in vivo*, the *in vivo* generation of azido groups of Apo-S-Ac_3_ManNAz-treated tumor tissues were monitored in PC-3 tumor-bearing mice model. To prepare PC-3 tumor-bearing mice model, PC-3 tumor cells were inoculated into both left and right flanks of nude mice (n = 5, 5 weeks old, male). When the tumor sizes reach 200–250 mm^3^, Apo-S-Ac_3_ManNAz (3αmg/kg) was intratumorally (I.T.) injected into both tumor tissues, once a day for four days. And then TRAIL (10 μg/kg) was directly I.T. injected into both left and right tumor tissues. As a control experiment, Cas-3 inhibitor (z-DEVD-FMK, 2 mg/kg) was directly I.T. injected to the left tumor tissues at 24 h before the last injection of Apo-S-Ac_3_ManNAz. For the bioorthogonal tracking of apoptosis *in vivo*, DBCO-Cy5.5 (4 mg/kg) was intravenously (I.V.) injected after 24 h post-injection of TRAIL to visualize the azido groups in tumor tissues via bioorthogonal click chemistry. Figure 5a showed the strong NIRF signal intensity of DBCO-Cy5.5 at the right tumor tissue (RT) at 24 h post-injection of DBCO-Cy5.5. However, the NIRF signal intensity of DBCO-Cy5.5 at the left tumor tissues (LT) was not noticeable at z-DEVD-FMK-treated LT. It is deduced that Apo-S-Ac_3_ManNAz successfully generated the azido groups on the tumor cell surface *in vivo* through Cas-3-specific metabolic engineering, caused by the TRAIL treatment. In addition, *ex vivo* NIRF images of tumor tissues showed that the NIRF intensity in RT was 1.68-fold higher than that of LT, pre-treated with z-DEVD-FMK (Fig. 5b and c). For histological analysis of the excised tumor tissues with immunofluorescence (IF) staining, the tumor tissues were sliced and stained with FITC-conjugated active Cas-3 antibody. And then fluorescence of Cas-3 and DBCO-Cy5.5 in tumor tissues was observed using CLSM. As expected, the strong fluorescence intensity of Cas-3 antibody was monitored in RT which was pre-treated with TRAIL, indicating that apoptosis was successfully induced in the tumor tissues. Importantly, the strong NIRF intensity of DBCO-Cy5.5 (red color) was co-localized with Cas-3 antibody (green color) in the right tumor tissue (RT), whereas z-DEVD-FMK-treated LT showed the minimum intensities both of DBCO-Cy5.5 and Cas-3 antibody (Fig. 5d and Figure S11). Finally, western blot data clearly showed that the strong azido groups-containing sialic acid bands were clearly observed on the RT, compared with z-DEVD-FMK treated LT (Fig. 5e). These results further supported that Apo-S-Ac_3_ManNAz could successfully generate the azido groups in the tumor tissues via Cas-3-specific cleavage reaction. Therefore, Apo-S-Ac_3_ManNAz-treated apoptotic tumor cells could be labeled with DBCO-Cy5.5 via bioorthogonal click chemistry and then could be monitored by non-invasive fluorescence imaging with a high-sensitivity *in vivo*.

**Figure 6:**
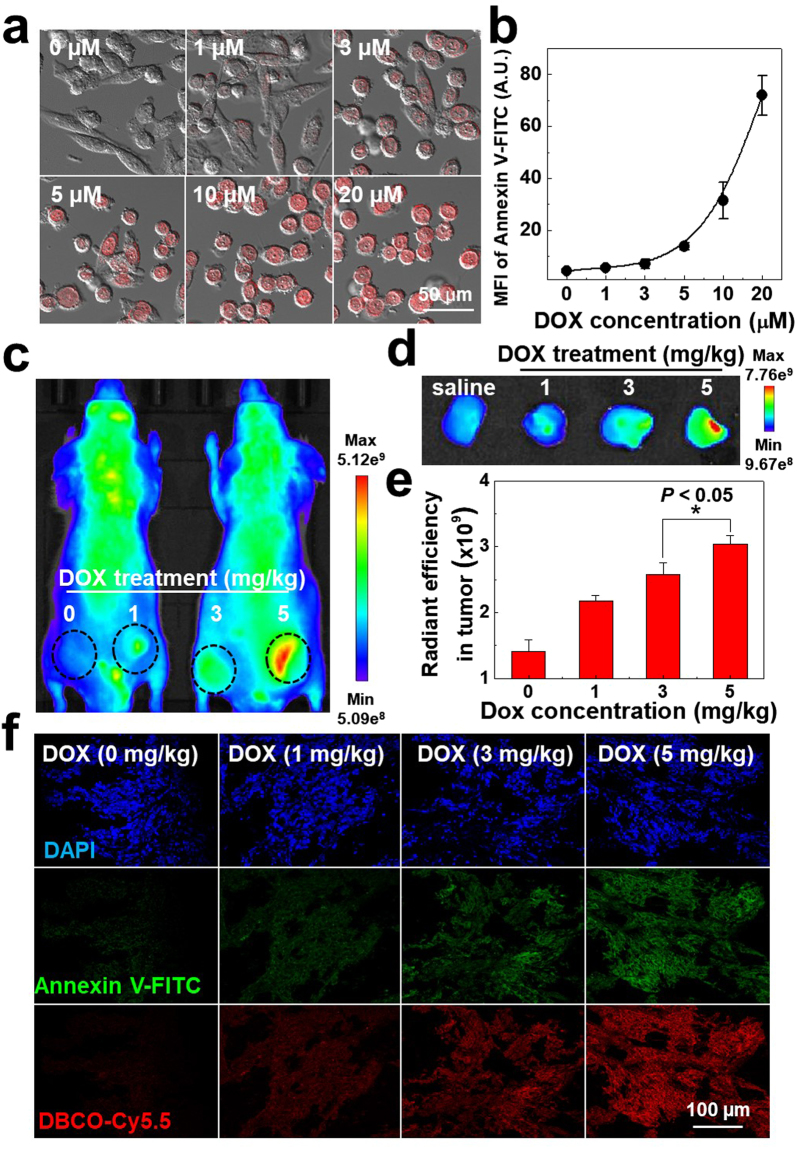
Non-invasive fast drug screening of doxorubicin (DOX) in Apo-S-Ac_3_ManNAz-treated PC-3 tumor-bearing mice (n = 5). (**a**) *In vitro* CLSM images of PC-3 tumor cells treated with different concentrations of DOX (0, 1, 3, 5, 10 and 20 μM) for 24 h. (**b**) FACS analysis of DOX-treated PC-3 tumor cells, wherein the apoptotic tumor cells were monitored using Annexin V-FITC staining. (**c**) *In vivo* non-invasive bioorthogonal apoptosis imaging of PC-3 tumor tissues after direct injection of DOX (0, 1, 3, 5 mg/kg) to PC-3 tumor-bearing mice. After 2 day post-injection of DOX, the apoptosis-induced azido groups were monitored with the I.V. injection of DBCO-Cy5.5 (4 mg/kg) and the NIRF intensity in each tumor tissue was quantitatively monitored using IVIS imaging system. (**d**) *Ex vivo* NIRF image of DOX-treated PC-3 tumor tissues (DOX’s concentration = 0, 1, 3, 5 mg/kg). (**e**) NIRF intensity of PC-3 tumor tissues (n = 5) was quantified by Living Image software. (**f**) CLSM images of PC-3 tumor tissues from the excised tumor tissues (**d**). (Red = DBCO-Cy5.5 channel; Green = Annexin V-FITC channel; Blue = DAPI channel).

Finally, to confirm the potential for the *in vivo* non-invasive fast drug screening using our bioorthogonal apoptosis tracking strategy, *in vivo* therapeutic efficacy of anticancer drug, doxorubicin (DOX), was monitored using PC-3 tumor-bearing mice. Firstly, *in vitro* cell culture system, the fluorescence intensity (DOX; red color) of DOX-treated PC-3 tumor cells greatly increased according to the DOX’s concentration from 1 to 20 μM, wherein DOX gradually localized in the nucleus part of PC-3 tumor cells after 24 h post-treatment of DOX and the DOX-treated cells exhibited a shrunken morphology in a dose-dependent manner, indicating the clear cell death (Fig. 6a). In addition, MFI of Annexin V-FITC-treated PC-3 tumor cells also gradually increased after treatment of 3 to 20 μM of DOX, indicating the DOX-derived cell death in cell culture system (Fig. 6b). Next, we evaluated *in vivo* therapeutic efficacy of DOX-treated tumor tissues using our bioorthogonal apoptosis tracking strategy in PC-3 tumor-bearing mice. In brief, Apo-S-Ac_3_ManNAz (3 mg/kg) was I.T. injected into the tumor tissues, once a day for four days. And then 1, 3, and 5 mg/kg of DOX were also I.T. injected to the each tumor tissue. For bioorthogonal tracking of apoptosis in tumor tissues, DBCO-Cy5.5 (4 mg/kg) was I.V. injected after 2 day post-injection of DOX. The NIRF intensity in tumor tissues were quantitatively monitored using IVIS imaging system. Interestingly, the NIRF intensity in tumor tissues gradually increased according to the DOX’s concentration, indicating that the strong NIRF signal of DBCO-Cy5.5 is closely related to the degree of apoptosis of DOX-treated PC-3 tumor cells (Fig. 6c). Furthermore, e*x vivo* NIRF image of the excised PC-3 tumor tissues also showed that the NIRF intensity proportionally increased according to the DOX’s concentration (Fig. 6d). The NIRF intensity of DBCO-Cy5.5 in the 1, 3 and 5 mg/kg of DOX-treated PC-3 tumor tissues were 1.54, 1.83 and 2.15-fold higher than non-treated tumor tissues, respectively (Fig. 6e). Importantly, the NIRF signal of DBCO-Cy5.5 in PC-3 tumor tissues was co-localized with that of Annexin V-FITC-treated PC-3 tumor tissues. Moreover, fluorescence intensity of DBCO-Cy5.5 and Annexin V-FITC (green color) gradually increased according to the DOX’s concentration, indicating that azido groups were successfully generated to the apoptotic PC-3 tumor cells not only *in vitro* cell culture condition but also *in vivo* tumor-bearing mice (Fig. 6f and Figure S12). Therefore, we expect that Apo-S-Ac_3_ManNAz and bioorthogonal click chemistry could be utilized for non-invasive apoptosis imaging for fast anticancer drug screening in tumor-bearing mice via the Cas-3/-7 activity based visualization of apoptosis.

Although repetitive intratumoral injection system of Apo-S-Ac_3_ManNAz might be impractical and various types of Cas-3/-7 activity-based imaging probes have been reported^36–38^, we developed Cas-3/-7-specific metabolic precursor for the first time, which could be utilized for bioorthogonal tracking of tumor apoptosis. Furthermore, in combination with various types of theranostic agents, such as superparamagnetic iron oxide nanoparticles (SPION), gold nanoparticles, liposomes and polymeric nanoparticles, azide groups on the apoptotic cell surface could be simply labeled via bioorthogonal click chemistry *in vitro* and *in vivo* ^39,40^. And then the labeled apoptotic cells will be able to monitor using various imaging tools, such as fluorescence imaging, magnetic resonance imaging (MRI), computed tomography (CT) and positron emission tomography (PET), with faster and higher spatiotemporal resolution. Thus we expect that Cas-3/-7-specific metabolic precursor, Apo-S-Ac_3_ManNAz, can be utilized for anticancer drug screening as well as apoptosis-based theranostic applications.

In summary, we have developed a Cas-3-specific metabolic precursor, Apo-S-Ac_3_ManNAz, which can be specifically cleaved into Ac_3_ManNAz by active Cas-3 in apoptotic tumor cells. The targetable azido groups (N_3_) were successfully generated on the cell surfaces, when we induced apoptosis using TRAIL (extrinsic apoptosis pathway) *in vitro* cell culture condition. Furthermore, azido groups on the apoptotic-cell surface successfully labeled with DBCO-Cy5.5 via bioorthogonal click chemistry *in vitro*. In addition, we also confirmed bioorthogonal tracking effect of Apo-S-Ac_3_ManNAz in both TRAIL and DOX (intrinsic apoptosis pathway)-induced *in vivo* apoptosis model. Importantly, Apo-S-Ac_3_ManNAz could provide bioorthogonal apoptosis tracking based on azido group generation to the anticancer drug-treated tumor tissues live animal models. Therefore, our Cas-3/-7-specific metabolic precursor with bioorthogonal click chemistry have potential for the bioorthogonal apoptosis imaging in living cells. Furthermore, our bioorthogonal apoptosis imaging strategy can markedly reduce costs and the time required for new drug discovery and therapeutic efficacy in cancer treatment.

## Materials and Methods

### Materials

The allyloxycarbonyl (Aloc) protected Cas-3 substrate peptide Ac-Lys(Aloc)-Gly-Asp(Aloc)-Glu(Aloc)-Val-Asp(Aloc) was purchased from Peptron (Daejeon, Korea). Triacetylated N-azidoacetyl-D-mannosamine (Ac_3_ManNAz) was purchased from FutureChem Co. Ltd (Seoul, Korea). Dibenzylcyclooctyne-Cy5.5 (DBCO-Cy5.5) was purchased from Click Chemistry Tools (Scottsdale, USA). Streptavidin-horseradish peroxidase (streptavidin-HRP) and Phosphine-PEG_3_-biotin were purchased from Thermo Fisher Scientific Inc. (Rockford, USA). N-ethoxycarbonyl-2-ethoxy-1,2-dihydroquinoline (EEDQ) and 4-aminobenzyl alcohol (4-AB-OH) were purchased from Tokyo Chemical Industry (Tokyo, Japan). Recombinant Human TRAIL/TNFSF10 protein, recombinant Human Cas-3 protein, Human/Mouse active Cas-3 antibody, Human/Mouse Cas-8 antibody, goat polyclonal GAPDH antibody, recombinant Human Cas-1, recombinant Human Cas-8, recombinant Human Cas-7, recombinant Human Cathepsin B and recombinant Human MMP-9 and N-benzyloxycarbonyl-Asp(OMe)-Glu(OMe)-Val-Asp(OMe)-fluoromethylketone (z-DEVD-FMK, Cas-3/-7 inhibitor) were purchased from R&D System (Minneapolis, USA). Dimethylformaide (DMF), trifluoroacetic acid (TFA), dimethyl sulfoxide (DMSO), 4-nitrophenyl chloroformate (4-NPC), N,N-dimethylpyridin-4-amine (DMAP), doxorubicin hydrochloride (DOX), Annexin-V assay kit and protease inhibitor cocktail were purchased from Sigma-Aldrich (St. Louis, USA). All the chemicals were analytical grade and used without further purification.

PC-3 (Human prostate adenocarcinoma) cell line was purchased from American Type Culture Collection (Rochkvile, USA). Dulbecco’s phosphate buffered saline (DPBS, pH 7.4), fetal bovine serum (FBS), RPMI 1640 media and antibiotics purchased from WelGENE Inc. (Daegu, Republic of Korea).

### Synthesis of AcKGDEVD-PABC-Ac_3_ManNAz (Apo-S-Ac_3_ManNAz)

First, a solution of Ac-Lys(Aloc)-Gly-Asp(Aloc)-Glu(Aloc)-Val-Asp(Aloc)-OH (652 mg, 0.45 mmol, Compound 1 in Figure S1) was mixed with 4-Aminobenzyl alcohol (113 mg, 0.9 mmol) and N-ethoxycarbonyl-2-ethoxy-1,2-dihydroquinoline (EEDQ; 220 mg, 1.75 mmol) in anhydrous dimethylformamide (DMF; 15 mL). Then, mixture was gently stirred for 24 h at room temperature under Ar (g). After reaction, DMF was evaporated by the rotatory evaporator and precipitated in diethyl ether to obtain Ac-Lys(Aloc)-Gly-Asp(Aloc)-Glu(Aloc)-Val-Asp(Aloc)-PABOH (compound 2 in Figure S1, Mass (ESI-MS, MW 1013.11): m/z 1035.23 [M+Na^+^]). Second, 4-nitrophenyl chloroformate (4-NPC, 29.7 mg, 0.15 mmol) and Ac-Lys(Aloc)-Gly-Asp(Aloc)-Glu(Aloc)-Val-Asp(Aloc)-PABOH (145 mg, 0.13 mmol) were dissolved in anhydrous dichloromethane (DCM; 2 mL). Then, 2,6-lutidine (60 µL, 0.6 mmol) was added to the reaction solution. After then, reaction solution was purified by high performance liquid chromatography (HPLC, Agilent Technologies 1200 series, Agilent Technologies, USA) to obtain Ac-Lys(Aloc)-Gly-Asp(Aloc)-Glu(Aloc)-Val-Asp(Aloc)-PABC (compound 3 in Figure S1, Mass (ESI-MS, MW 1178.22): m/z 1200.34 [M+Na^+^]). In the next step, N,N-dimethylpyridin-4-amine (DMAP; 6.2 mg, 0.05 mmol) and Ac_3_ManNAz (10.8 mg, 0.03 mmol) were mixed with the solution of Ac-Lys(Alloc)-Gly-Asp(Alloc)-Glu(Alloc)-Val-Asp(Alloc)-PABC (31 mg, 0.025 mmol) in DCM (3 mL). Then, the reaction solution was stirred for 12 h at room temperature and purification was performed by HPLC to obtain Ac-Lys(Alloc)-Gly-Asp(Alloc)-Glu(Alloc)-Val-Asp(Alloc)-PABC-Ac_3_ManNAz (compound 4 in Figure S1, Mass (ESI-MS, MW 1427.44): m/z 1449.56 [M+Na^+^]). For the deprotection of compound 4, tetrakis(triphenylphosphine)palladium (Pd(PPh_3_)_4_; 6.7 mg, 0.006 mmol) and Ac-Lys(Alloc)-Gly-Asp(Alloc)-Glu(Alloc)-Val-Asp(Alloc)-Ac_3_ManNAz (17.5 mg, 0.023 mmol) were dissolved in DMF (2 mL). And then acetic acid (13.3 µL, 0.232 mmol) and tributyltin hydride (Bu_3_SnH; 53 µL, 0.2 mmol) were added to reaction mixture and stirred for 1 h at room temperature. Finally, the residue was removed by HPLC to obtain Ac-Lys-Gly-Asp-Glu-Val-Asp-PABC-Ac_3_ManNAz (compound 5 in Figure S1, Apo-S-Ac_3_ManNAz, Mass (ESI-MS, MW 1223.3): m/z 1245.42 [M+Na^+^]). To confirm of chemical structures of Apo-S-Ac_3_ManNAz, it was freshly dissolved in DMSO-d_6_ and characteristic peaks were monitored by 600 MHz ^1^H-NMR (DD2 600 MHz FT NMR, Agilent Technologies, USA). The chemical structure of Apo-S-Ac_3_ManNAz was analyzed using characteristic peaks at 4.5 ppm (-CH at Lys and Asp), 2.02 ppm (-CH_3_ at Ac_3_ManNAz), 7.38 and 7.56 ppm (-CH at PABC linker) (Figure S2). The molecular weight of Apo-S-Ac_3_ManNAz was further confirmed using matrix-assisted laser desorption/ionization (MALDI) analysis with cyano-4-hydroycinnaminc acid matrix (AB Sciex TOF/TOF 5800 System, USA). The exact mass of Apo-S-Ac_3_ManNAz was verified to 1223.44 [M+H^+^], 1245.42 [M+Na^+^] and 1267.4 [M+2Na^+^] m/z, respectively (Figure S3).

### ***In vitro*enzyme-specific release of Ac**_**3**_**ManNAz from Apo-S-Ac**_**3**_**ManNAz**

To observe Cas-3/-7-specific release of Ac_3_ManNAz from Apo-S-Ac_3_ManNAz, 1 mM of Apo-S-Ac_3_ManNAz was incubated with Cas-3 or Cas-7 enzyme (15 µg/mL) in a caspase-activation buffer (200 mM HEPES, pH 7.4, 1% CHAPS and 20 mM EDTA), respectively, for 0, 3, 6 and 24 h at 37 °C. The samples were analyzed using HPLC system (Agilent Technologies 1200 series, Agilent Technologies, USA) equipped with C18 analytical column (0: 100 Acetonitrile +0.1% TFA: H_2_O to 40: 60 Acetonitrile +0.1% TFA: H_2_O for 1 h), monitoring at 230 nm (Fig. 2a and Figure S4a). As a control experiment, 15 µg/mL of scrambled enzyme (Cas-1, Cas-8, MMP-9, Cathepsin B) were incubated with Apo-S-Ac_3_ManNAz for 24 h at 37 °C, and the samples were analyzed using HPLC system as described above (Fig. 2b). Hydrolysis effect of Apo-S-Ac_3_ManNAz *in vitro* was monitored by HPLC system using Apo-S-Ac_3_ManNAz which directly incubated without Cathepsin B in DPBS at 37 °C. At 0, 3, 6 and 24 h post-incubation, the samples were analyzed by HPLC system as described above (Figure S4b).

### Cell viability and proliferation assays

PC-3 tumor cells were cultured in RPMI 1640 medium containing 10% (v/v) FBS and 1% (v/v) penicillin–streptomycin at 37 ^◦^C in a humidified 5% CO_2_ incubator. The cytotoxicity of Apo-S-Ac_3_ManNAz was monitored using the MTT assay (Figure S6a). In brief, PC-3 tumor cells were seeded at 5 × 10^3^ cells/well in 96-well plate and stabilized for 24 h. After stabilizing, PC-3 tumor cells were incubated for 24 h with various concentrations of the Apo-S-Ac_3_ManNAz (0, 1, 5, 10, 20, 50, and 100 µM). Then, 25 µL of the MTT solution containing RPMI 1640 media (100 µl) was added to each well, and PC-3 tumor cells were incubated for an additional 30 min at 37 °C. MTT solution containing RPMI 1640 media was removed and PC-3 tumor cells were incubated with 200 µL of DMSO for 20 min. The absorbance of each well was measured at 562 nm using a microplate reader (VERSAmaxTM, Molecular Devices Corp., Sunnyvale, CA). In addition, proliferation of Apo-S-Ac_3_ManNAz (20 µM) treated PC-3 tumor cells was monitored using the CCK-8 assay (Dojindo Molecular Technologies, Inc., Rockvile, USA) for 1, 2, and 3 days (Figure S6b). In brief, PC-3 tumor cells were seeded at 5 × 10^3^ cells/well in 96-well plate and stabilized for 24 h. After stabilizing, PC-3 tumor cells were incubated for 1, 2, and 3 days with 20 µM of the Apo-S-Ac_3_ManNAz. And then, 10 µL of the CCK-8 solution was added to each well, and PC-3 tumor cells were incubated for an additional 40 min at 37 °C. The absorbance of each well was measured at 450 nm using a microplate reader.

Cell viability of PC-3 tumor cells treated with TRAIL was measured by MTT assay (Figure S6c). In brief, PC-3 tumor cells were seeded at 5 × 10^3^ cells/well in 96-well plate and stabilized for 24 h. After stabilization, PC-3 tumor cells were washed with DPBS and incubated for 24 h with various concentrations of TRAIL (0, 5, 10, 25, 50, 100, and 200 ng/ml). Then, 25 µL of the MTT solution containing RPMI 1640 media (100 µl) was added to each well, and PC-3 tumor cells were incubated for an additional 30 min at 37 °C. MTT solution containing RPMI 1640 media was removed and PC-3 tumor cells were incubated with 200 µL of DMSO for 20 min. The absorbance of each well was measured at 562 nm using a microplate reader as described above. The morphological changes of PC-3 tumor cell which treated with 7 ng/ml of TRAIL were monitored at 0, 1, 3, 6, 9, and 24 h using optical microscope (CK40, Olympus, Japan) (Figure S6d).

### Western blot analysis for activation of Cas-3 by TRAIL treatment

To observe activation of Cas-3 by TRAIL treatment, 3 × 10^4^ cells of PC-3 tumor cells were seeded into 6-well plate with RPMI 1640 media for 48 h at 37 °C in a humidified 5% CO_2_ incubator. Then, 7 ng of TRAIL was added to RPMI 1640 media and the PC-3 tumor cells were further incubated for 0, 1, 3, 6, 9, and 24 h at 37 °C. After incubation, the PC-3 tumor cells were washed with DPBS and were lysed using RIPA buffer (Thermo Fisher Scientific Inc., USA) with 1% protease inhibitor cocktail. Lysates were centrifuged at 12,000 rpm for 20 min at 4 °C to remove cell debris. The total protein of each lysate was quantified by BCA assay (Pierce^®^ BCA Protein Assay Kit, Thermo Scientific Inc., USA). Then, 1 x sodium dodecyl sulfate gel-loading dye (2% SDS, 5% glycerol, 125 mol/L Tris pH 6.8, 0.006% bromophenol blue and 1% mercaptoethanol) and boiled for 5 min. 20 µg of proteins were separated by 12% SDS-polyacrylamide gel electrophoresis and transferred onto PVDF membranes for 90 min at 120 V. The membranes were incubated with 4% bovine serum albumin (BSA) containing TBST solution (10 mol/L Tris, pH 7.4, 100 mol/L NaCl and 0.1% Tween 20) for 1 h at room temperature. Then, the membranes were incubated with 0.2 µg/mL of goat anti-human Cas-8 antibody or 0.2 µg/mL of goat anti-human active Cas-3 antibody or 1 µg/mL goat polyclonal GAPDH antibody containing 4% BSA-TBST solution (10 mol/L Tris, pH 7.4, 100 mol/L NaCl and 0.1% Tween 20) for 12 h at 4 °C. To visualization of each protein, the membranes were washed 5 times by TBST solution, and then incubated with 0.2 µg/mL of mouse anti-goat IgG-HRP antibody containing 4% BSA-TBST solution for 2 h at room temperature. After 3 more washes, Cas-8, active-Cas-3, and GAPDH protein band were detected with an ECL system (Fig. 3a).

### Western blot analysis for azido (N_3_) groups-containing sialic acid in PC-3 tumor cells

To separation and visualization of azido groups-containing sialic acid, each lysate from PC-3 tumor cells was analyzed by western blot analysis as described previous report^1^. To observe non-specific generation of azido (N_3_) groups in healthy PC-3 tumor cells, 3 × 10^4^ of PC-3 tumor cells were seeded into 6-well plate with 20 µM of Apo-S-Ac_3_ManNAz or 20 µM of Ac_3_ManNAz containing RPMI 1640 media for 0, 1, 3, 6, 9, and 24 h at 37 °C (Fig. 2d). Next, we observed azido groups generation of PC-3 tumor cells after treatment of Apo-S-Ac_3_ManNAz and TRAIL. In brief, 3 × 10^4^ of PC-3 tumor cells were seeded into 6-well plate with 20 µM of Apo-S-Ac_3_ManNAz containing RPMI 1640 media for 4 h at 37 °C. And then, 7 ng of TRAIL was added to each media and PC-3 tumor cells were further incubated for 0, 1, 3, 6, 9, and 24 h at 37 °C (Fig. 3b). As a control experiment, 200 µM of z-DEVD-FMK which an irreversible Cas-3 inhibitor was treated to PC-3 tumor cells for 24 h before treatment of Apo-S-Ac_3_ManNAz and TRAIL. In brief, 3 × 10^4^ of PC-3 tumor cells were seeded into 6-well plate with 200 µM of z-DEVD-FMK and then incubated for 24 h at 37 °C. To generation of azido groups, the PC-3 tumor cells were incubated with 20 µM of Apo-S-Ac_3_ManNAz containing RPMI 1640 media for 4 h at 37 °C. And then, 7 ng of TRAIL was added to each media and PC-3 tumor cells were further incubated for 24 h at 37 °C (Fig. 3g). For western blot analysis, the PC-3 tumor cells were washed twice with DPBS and were lysed using RIPA buffer with 1% protease inhibitor. Each lysate was centrifuged at 14,000 rpm for 20 min at 4 °C to removed cell debris. The total protein of each lysate was quantified by BCA assay and the lysates were incubated with 500 nM of phosphine–PEG_3_-biotin (Pierce, Rockford, IL, USA) for 6 h at 37 °C. The proteins from each sample were mixed with 1 x sodium dodecyl sulfate (SDS) gel-loading buffer and boiled for 5 min. 20 μg of proteins were separated by 10% SDS-polyacrylamide gel electrophoresis and transferred onto PVDF membranes. The membranes were blocked for 1 h at room temperature in 5% bovine serum albumin (BSA) containing 1 x TBST solution (10 mol/L Tris, pH 7.4, 100 mol/L NaCl and 0.1% Tween 20). Then the membranes were incubated with streptavidin-HRP (Pierce, Rockford, IL, USA) containing 1 x TBST solution for 12 h at 4 °C. Finally, the membranes were washed three times using 1 x TBST and protein band was detected with an ECL system. As the control group, the total protein was visualized by Comassies brilliant blue stain.

### ***In vitro*bioorthogonal apoptosis tracking using Apo-S-Ac**_**3**_**ManNAz-treated PC-3 tumor cells**

To observe non-specific generation of azido (N_3_) groups by Apo-S-Ac_3_ManNAz, 3 × 10^4^ of PC-3 tumor cells were seeded into 35 mm cover glass bottom dishes with 20 µM of Apo-S-Ac_3_ManNAz or 20 µM of Ac_3_ManNAz containing RPMI 1640 media for 0, 1, 3, 6, 9, and 24 h at 37 °C (Fig. 2c and Figure S5). To visualization of azido groups on the PC-3 tumor cells, the PC-3 tumor cells were washed twice with DPBS and then they were incubated with 5 µM of DBCO-Cy5.5 containing RPMI 1640 media for 1 h at 37 °C. Then, the PC-3 tumor cells were washed twice with DPBS and fixed using fixation buffer (DPBS containing 10% formaldehyde, 1% glutaraldehyde) for 20 min at room temperature. After fixation, the PC-3 tumor cells were stained with DAPI for 10 min at room temperature. Fluorescence and morphology of the PC-3 tumor cells were observed by confocal laser scanning microscope (CLSM) with 405 diode (405 nm), Ar (458, 488, 514 nm) and He-Ne (633 nm) lasers (Leica TCS SP8, Leica Microsystems GmbH, Germany). To observe Cas-3-specific generation of azido (N_3_) groups by Apo-S-Ac_3_ManNAz, 3 × 10^4^ of PC-3 tumor cells were seeded into 35 mm cover glass bottom dishes with 20 µM of Apo-S-Ac_3_ManNAz containing RPMI 1640 media for 4 h at 37 °C. Thereafter, 7 ng of TRAIL was added to each media and PC-3 tumor cells were further incubated for 0, 1, 3, 6, 9, and 24 h at 37 °C (Fig. 3c and Figure S8a). To visualization of azido groups on the PC-3 tumor cells, the PC-3 tumor cells were washed twice with DPBS and then they were incubated with 5 µM of DBCO-Cy5.5 containing RPMI 1640 media for 1 h at 37 °C. Then, the PC-3 tumor cells were washed twice with DPBS and fixed using fixation buffer (DPBS containing 10% formaldehyde, 1% glutaraldehyde) for 20 min at room temperature. After fixation, the PC-3 tumor cells were stained with DAPI for 10 min at room temperature. Fluorescence and morphology of the PC-3 tumor cells were observed by CLSM as described above. For the quantification of DBCO-Cy5.5 labelled PC-3 tumor cells, we performed flow cytometry (FACS) analysis based on the fluorescence intensity of the Apo-S-Ac_3_ManNAz- and TRAIL-treated PC-3 tumor cells (Fig. 3d). In brief, 3 × 10^4^ of PC-3 tumor cells were seeded into 6-well plate with 20 µM of Apo-S-Ac_3_ManNAz containing RPMI 1640 media for 4 h at 37 °C. Thereafter, 7 ng of TRAIL was added to each well plates and PC-3 tumor cells were further incubated for 0, 1, 3, 6, 9, and 24 h at 37 °C. Then, the PC-3 tumor cells were washed twice with DPBS and incubated with 5 µM of DBCO-Cy5.5 for 1 h at 37 °C. The PC-3 tumor cells were washed twice with DPBS and collected in 2% FBS contained DPBS and then the fluorescence of the DBCO-Cy5.5 in the PC-3 tumor cells was analyzed by FACS analysis (Guava easyCyte^TM^, Merck Millipore, Germany). To observe Cas-3-specific generation of azido (N_3_) groups on the LNCaP and HT-29 tumor cells by Apo-S-Ac_3_ManNAz, 3 × 10^4^ of LNCaP and HT-29 tumor cells were seeded into 35 mm cover glass bottom dishes with 20 µM of Apo-S-Ac_3_ManNAz containing RPMI 1640 media for 4 h at 37 °C. Thereafter, 7 ng of TRAIL was added to each media and LNCaP and HT-29 tumor cells were further incubated for 24 h at 37 °C. To visualization of azido groups on the LNCaP and HT-29 tumor cells, LNCaP and HT-29 tumor cells were washed twice with DPBS and then they were incubated with 5 µM of DBCO-Cy5.5 containing RPMI 1640 media for 1 h at 37 °C. Then, the LNCaP and HT-29 tumor cells were washed twice with DPBS and fixed using fixation buffer (DPBS containing 10% formaldehyde, 1% glutaraldehyde) for 20 min at room temperature. After fixation, the LNCaP and HT-29 tumor cells were stained with DAPI for 10 min at room temperature. Fluorescence of the LNCaP and HT-29 tumor cells were observed by CLSM (Figure S9a and S9c). Fluorescence intensity of LNCaP and HT-29 tumor cells were quantified using Image J software (Figure S9b and 9d).

As a control experiment, PC-3 cells were treated with z-DEVD-FMK as an irreversible Cas-3 inhibitor to inhibition of Cas-3 activity before treatment of Apo-S-Ac_3_ManNAz and TRAIL. In brief, 3 × 10^4^ of PC-3 tumor cells were seeded into 35 mm cover glass bottom dishes with 200 µM of z-DEVD-FMK and then incubated for 24 h at 37 °C. To evaluation of generating azido groups, the PC-3 tumor cells were incubated with 20 µM of Apo-S-Ac_3_ManNAz containing RPMI 1640 media for 4 h at 37 °C. And then, 7 ng of TRAIL was added to each media and PC-3 tumor cells were further incubated for 24 h at 37 °C (Fig. 3e and Figure S8b). To visualization of azido groups on the PC-3 tumor cells, the PC-3 tumor cells were washed twice with DPBS and then they were incubated with 5 µM of DBCO-Cy5.5 containing RPMI 1640 media for 1 h at 37 °C. Then, the PC-3 tumor cells were washed twice with DPBS and fixed using fixation buffer (DPBS containing 10% formaldehyde, 1% glutaraldehyde) for 20 min at room temperature. After fixation, the PC-3 tumor cells were stained with DAPI for 10 min at room temperature. Fluorescence and morphology of the PC-3 tumor cells were observed by CLSM as described above. For the quantification of DBCO-Cy5.5 labelled PC-3 tumor cells, we performed FACS analysis as described above (Fig. 3f).

Finally, bioorthogonal apoptosis tracking of Apo-S-Ac_3_ManNAz-treated PC-3 tumor cells was further analyzed using Annexin V-FITC apoptosis detection kit (Sigma Aldrich, USA). In brief, 3 × 10^4^ of the PC-3 tumor cells were seeded into 35 mm cover glass bottom dishes with 20 µM of Apo-S-Ac_3_ManNAz containing RPMI 1640 media for 4 h at 37 °C. Then, 7 ng of TRAIL was added to each media and the PC-3 tumor cells were further incubated for 6, 9, and 24 h at 37 °C. To visualization of azido groups on the PC-3 tumor cells, the PC-3 tumor cells were washed twice with DPBS and then they were incubated with 5 µM of DBCO-Cy5.5 containing RPMI 1640 media for 1 h at 37 °C. Then, the PC-3 tumor cells were incubated with Annexin V-FITC (5 µl) and propidium iodide (PI, 10 µl) containing binding buffer (1.4 M NaCl and 25 mM CaCl_2_ containing 100 mM HEPES/NaOH, pH 7.5) for 10 min at room temperature. Fluorescence of the PC-3 tumor cells were observed by CLSM as described above (Fig. 4a). For the quantification of DBCO-Cy5.5, Annexin V-FITC, and PI in the PC-3 tumor cells, the PC-3 tumor cells were washed twice with DPBS and collected in 2% FBS contained DPBS. And then, fluorescence of the DBCO-Cy5.5, Annexin V-FITC and PI in the PC-3 tumor cells was analyzed by FACS analysis (Fig. 4b).

### ***In vivo*bioorthogonal apoptosis tracking using PC-3 tumor-bearing mice**

All experiments with live animals were performed in compliance with the relevant laws and institutional guidelines of Institutional Animal Care and Use Committee (IACUC) in Korea Institute of Science and Technology (KIST), and IACUC approved the experiment (approved number of 2017-005). Athymic nude mice (5 weeks old, 20–25 g, male) was purchased from Nara Bio INC. (Gyeonggi-do, Korea). To prepare PC-3 tumor-bearing mice model, a suspension of 5 × 10^6^ of PC-3 tumor cells in RPMI 1640 media (80 µl) was injected into the both of left and right flanks of mice. When the PC-3 tumor tissue grew up approximately 200–250 mm^3^ in volume, Apo-S-Ac_3_ManNAz (3 mg/kg) was directly injected (I.T.) into the both of left and right tumor tissues, once a day for 4 days. In addition, z-DEVD-FMK (2 mg/kg) was directly injected (I.T.) into the left tumor tissue to inhibit Cas-3 activity. At 24 h post-injection of z-DEVD-FMK, 10 µg/kg of TRAIL was directly injected (I.T.) into the both left and right tumors for inducing apoptosis of the tumor tissues. At 24 h post-treatment of TRAIL, DBCO-Cy5.5 (4 mg/kg) was intravenously injected (I.V.) to the PC-3 tumor-bearing mice (n = 5) via tail vein and NIRF intensity from PC-3 tumor-bearing mice was monitored using IVIS Lumina Series III (PerkinElmer, USA) at 48 h post-injection of DBCO-Cy5.5 (Fig. 5a). To analysis of *ex vivo* NIRF intensity in tumor tissues, PC-3 tumor-bearing mice was sacrificed at 48 h post-injection of DBCO-Cy5.5. Both left and right tumor tissues were excised and then NIRF intensity of tumor tissues was monitored by IVIS Lumina Series III (Fig. 5b). NIRF intensity in tumor ROIs was quantified using Living Image software (PerkinElmer, USA) (Fig. 5c).

The Cas-3 activation in excised PC-3 tumor tissues was observed using immunofluorescence (IF) stain with active Cas-3 antibody (Fig. 5d and Figure S11). Briefly, dissected tumor tissues were retrieved from PC-3 tumor-bearing mice, fixed in OCT compound and freezed for 24 h at −20 °C. Then, the tissue block were sectioned at 10 µm. For active Cas-3 staining, the tissue slides were washed with PBS (pH 7.4) for three times and blocking solution (4% bovine serum albumin containing PBS (pH 7.4)) for 1 h at room temperature. Then, the tissue slides were washed with PBS (pH 7.4) for three times and incubated with active Cas-3 antibody (0.2 µg/mL, 4% BSA containing PBS (pH 7.4)) for 12 at 4 °C. To visualization of active Cas-3, FITC-conjugated rabbit anti-mouse IgG antibody (1:500, 4% BSA containing PBS (pH 7.4)) was treated to the tissue slides for 2 h at room temperature. Finally, the tissue slides were washed with PBS (pH 7.4) for three times and mounted using cover glass. The fluorescence in the PC-3 tumor tissue was observed using a confocal laser microscope (Leica TCS SP8, Leica Microsystems GmbH, Germany) with 405 diode (405 nm), Ar (458, 488, 514 nm) and He-Ne (633 nm) lasers.

To observe azido groups generation in the PC-3 tumor tissues, PC-3 tumor-bearing mice model was prepared as described above. In brief, a suspension of 5 × 10^6^ PC-3 cells in RPMI 1640 media (80 µl) was injected into the both of left and right flanks of mice. When the PC-3 tumor tissue grew up approximately 200–250 mm^3^ in volume, Apo-S-Ac_3_ManNAz (3 mg/kg) was directly injected (I.T.) into the both of left and right tumor tissues, once a day for 4 days. In addition, z-DEVD-FMK (2 mg/kg) was directly injected (I.T.) into the left tumor tissue to inhibit Cas-3 activity. At 24 h post-injection of z-DEVD-FMK, 10 µg/kg of TRAIL was directly injected (I.T.) into the both left and right tumors for inducing apoptosis of the tumor tissues. At 24 h post-treatment of TRAIL, the PC-3 tumor tissues were dissected and lysed using RIPA buffer with 1% protease inhibitor. Total protein in each lysate was quantified by BCA assay and the lysates were incubated with 500 nM of phosphine-PEG_3_-biotin for 6 h at room temperature. The lysates were mixed with 1 x sodium dodecyl sulfate (SDS) gel-loading buffer and boiled for 5 min. 10 µg of proteins were separated by 12% SDS-polyacrylamide gel electrophoresis and transferred onto PVDF membranes for 90 min at 120 V. The membranes were blocked for 1 h at room temperature in 5% bovine serum albumin (BSA) containing 1 x TBST solution (10 mol/L Tris, pH 7.4, 100 mol/L NaCl and 0.1% Tween 20). Then the membranes were incubated with streptavidin-HRP (Pierce, Rockford, IL, USA) containing 1 x TBST solution for 12 h at 4 °C. Finally, the membranes were washed three times using 1 x TBST and protein band was detected with an ECL system. As the control group, the total protein was visualized by Comassies brilliant blue stain (Fig. 5e).

### ***In vivo*bioorthogonal apoptosis tracking for fast drug screening analysis**

To observe the potential for the *in vivo* non-invasive fast drug screening using our bioorthogonal apoptosis tracking strategy, doxorubicin hydrochloride (DOX) was utilized as a model anticancer drug. First, we monitored DOX-induced apoptosis of PC-3 tumor cells in the cell culture system. Briefly, 5 × 10^4^ of PC-3 tumor cells were seeded into 35 mm cover glass bottom dishes and stabilized for 48 h. After stabilization, the PC-3 tumor cells were incubated with various concentrations of DOX (0, 1, 3, 5, 10, and 20 µM) for 24 h at 37 °C. Then, the PC-3 tumor cells were washed twice with DPBS and fixed using fixation buffer (DPBS containing 10% formaldehyde, 1% glutaraldehyde) for 20 min at room temperature. After fixation, fluorescence and morphology of the PC-3 tumor cells were observed by CLSM with 405 diode (405 nm), Ar (458, 488, 514 nm) and He-Ne (633 nm) lasers (Leica TCS SP8, Leica Microsystems GmbH, Germany) (Fig. 6a). In addition, DOX-induced apoptosis of the PC-3 tumor cells was further analyzed using Annexin V-FITC. In brief, 5 × 10^4^ of PC-3 tumor cells were seeded into 6-well plate and stabilized for 48 h. After stabilization, the PC-3 tumor cells were incubated with various concentrations of DOX (0, 1, 3, 5, 10, and 20 µM) for 24 h at 37 °C. After DOX treatment, the PC-3 tumor cells were incubated with Annexin V-FITC (5 µl) containing binding buffer (1.4 M NaCl and 25 mM CaCl_2_ containing 100 mM HEPES/NaOH, pH 7.5) for 10 min at room temperature. Then, the PC-3 tumor cells were washed twice with DPBS and collected in 2% FBS contained DPBS. The fluorescence of the Annexin V-FITC in the PC-3 tumor cells was analyzed by FACS analysis (Guava easyCyte^TM^, Merck Millipore, Germany) (Fig. 6b).

Next, we observed non-invasive fast drug screening using our bioorthogonal apoptosis tracking strategy, 5 × 10^6^ of PC-3 tumor cells were directly injected both of left and right flanks of mice. When tumor grew up approximately 200–250 mm^3^, Apo-S-Ac_3_ManNAz (3 mg/kg) were directly injected to tumors (I.T.) once a day for 4 days. After 24 h post-injection of Apo-S-Ac_3_ManNAz, various dose of DOX (0, 1, 3, and 5 mg/kg) were directly injected (I.T.) to the PC-3 tumor tissues. At 48 h post-injection of DOX, DBCO-Cy5.5 (4 mg/kg) was intravenously injected (I.V.) to the PC-3 tumor-bearing mice (n = 5) via tail vein and NIRF intensity from PC-3 tumor-bearing mice was monitored using IVIS Lumina Series III (PerkinElmer, USA) at 48 h post-injection of DBCO-Cy5.5 (Fig. 6c). To analysis of *ex vivo* NIRF intensity in tumor tissues, PC-3 tumor-bearing mice was sacrificed at 48 h post-injection of DBCO-Cy5.5. The PC-3 tumor tissues were excised and then NIRF intensity of DBCO-Cy5.5 in tumor tissues was monitored by IVIS Lumina Series III (Fig. 6d). NIRF intensity in tumor ROIs was quantified using Living Image software (PerkinElmer, USA) (Fig. 6e).

For the tissue fluorescence analysis of DOX-treated PC-3 tumor tissues, the PC-3 tumor tissues which were 0, 1, 3, and 5 mg/kg DOX-treated were sectioned at 20 µm. For observation of apoptosis in the PC-3 tumor tissues, tumor tissue slides were washed with PBS (pH 7.4) for three times and incubated with Annexin V-FITC (10 µl) containing binding buffer (1.4 M NaCl and 25 mM CaCl_2_ containing 100 mM HEPES/NaOH, pH 7.5) for 15 min. And then, tissue slides were washed with PBS (pH 7.4) for three times and mounted using cover glass. The fluorescence in the tumor tissue was observed using a CLSM as described above (Fig. 6f and Figure S12).

### Statistics

In this study, the differences between experimental and control groups were analyzed using one-way ANOVA and considered statistically significant (marked with an asterisk (*) in figure).

## Electronic supplementary material

Supporting information
