# Supplementary material for: Caspase-3/-7-Specific Metabolic Precursor for Bioorthogonal Tracking of Tumor Apoptosis
Source: Sci Rep. 2017 Nov 30;7:16635. doi: 10.1038/s41598-017-16653-2 (PMC5709468; doi:10.1038/s41598-017-16653-2)
Supplement: Supplementary file 1 — Supporting information [file 41598_2017_16653_MOESM1_ESM.pdf]

<*Supplementary information for Scientific Reports*>

Caspase-3/-7-Specific Metabolic Precursor for Bioorthogonal Tracking of Tumor Apoptosis

Man Kyu Shim[1‡], Hong Yeol Yoon[2‡], Sangmin Lee[3], Mun Kyeong Jo[2,4], Jooho Park[2], Jong-Ho Kim[1], Seo Young Jeong[4], Ick Chan Kwon[2,5] and Kwangmeyung Kim[2,5]*

[1] Department of Pharmacy, Graduate School, Kyung Hee University, 26, Kyungheedae-ro, Dongdaemun-gu, Seoul 02447, Republic of Korea

[2] Center for Theragnosis, Biomedical Research Institute, Korea Institute of Science and Technology, 5, Hwarang-ro 14-gil, Seongbuk-gu, Seoul 02792, Republic of Korea

[3] Department of Pharmacy, College of Pharmacy, Wonkwang University, 460 Iksandae-ro, Iksan, Jeonbuk 54538, Republic of Korea

[4] Department of Life and Nanopharmaceutical Science, Kyung Hee University, 26, Kyungheedae-ro, Dongdaemun-gu, Seoul 02447, Republic of Korea

[5] KU-KIST Graduate School of Converging Science and Technology, Korea University, 145 Anam-ro, Seongbuk-gu, Seoul, 02841, Republic of Korea

* Corresponding authors: Kwangmeyung Kim (kim@kist.re.kr)

‡ These authors have equally contributed.

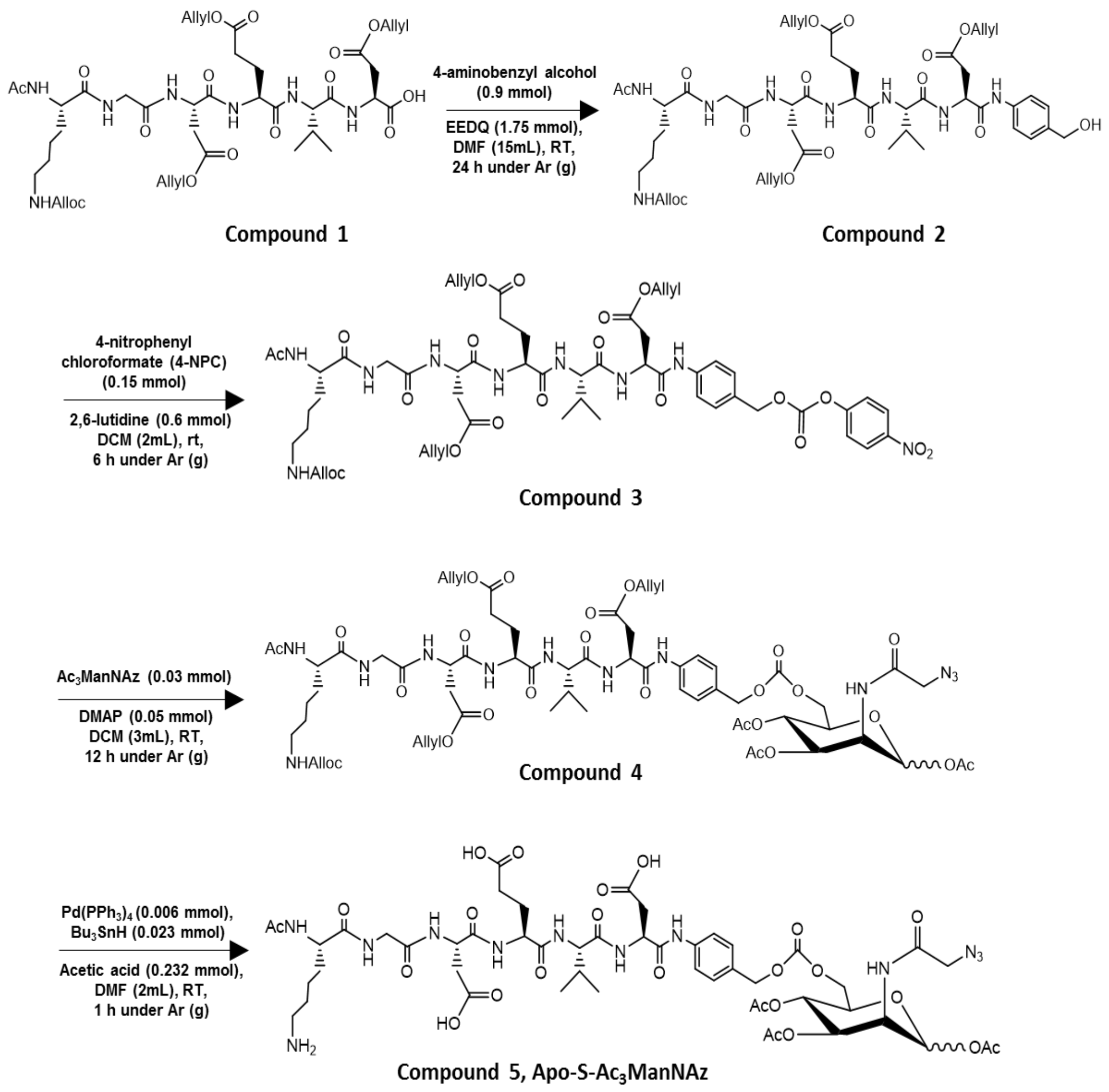

Figure S1. Synthetic scheme of Apo-S-Ac_3ManNAz.

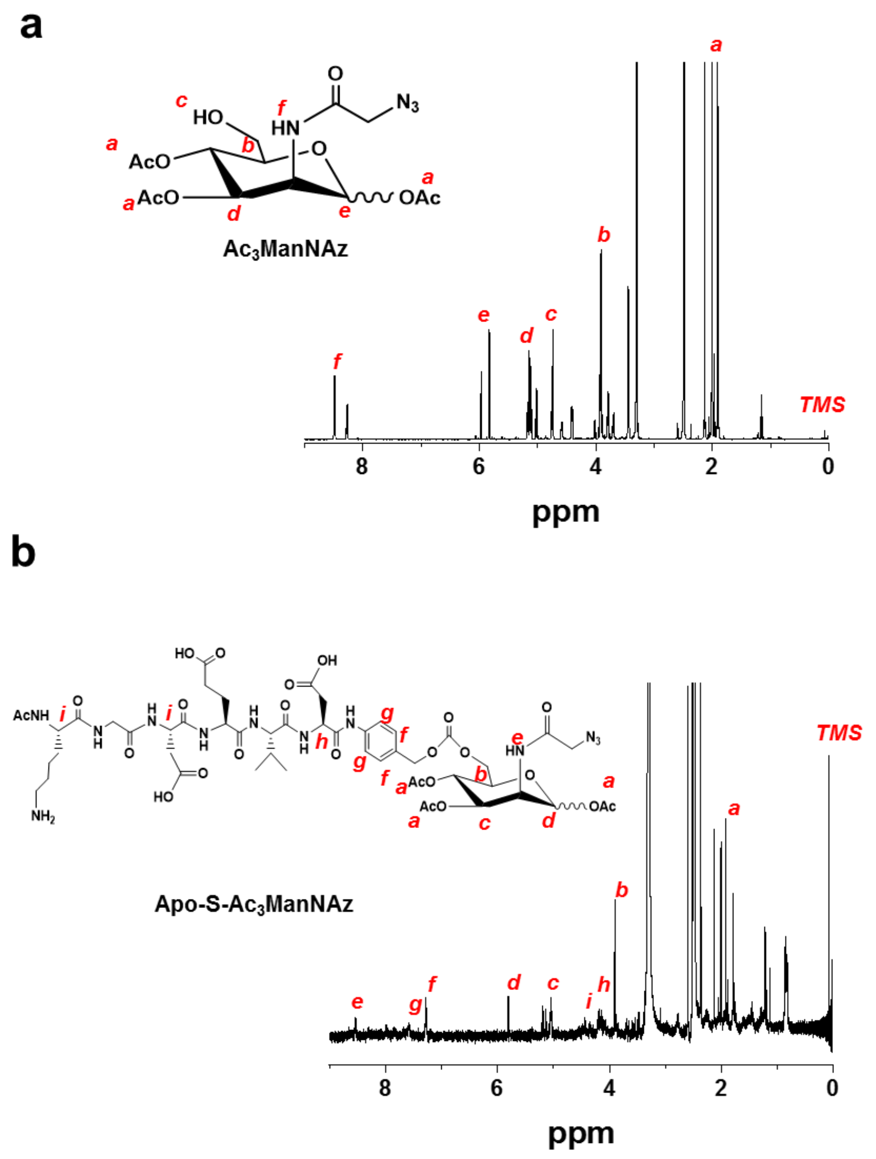

Figure S2. 600MHz ^{1}H-NMR spectrum of (a) Ac$_3$ManNAz (mixed anomers $\alpha:\beta = 1:3$) and (b) Apo-S-Ac$_3$ManNAz (mixed anomers $\alpha:\beta = 1:3$) in DMSO-d$_6$.

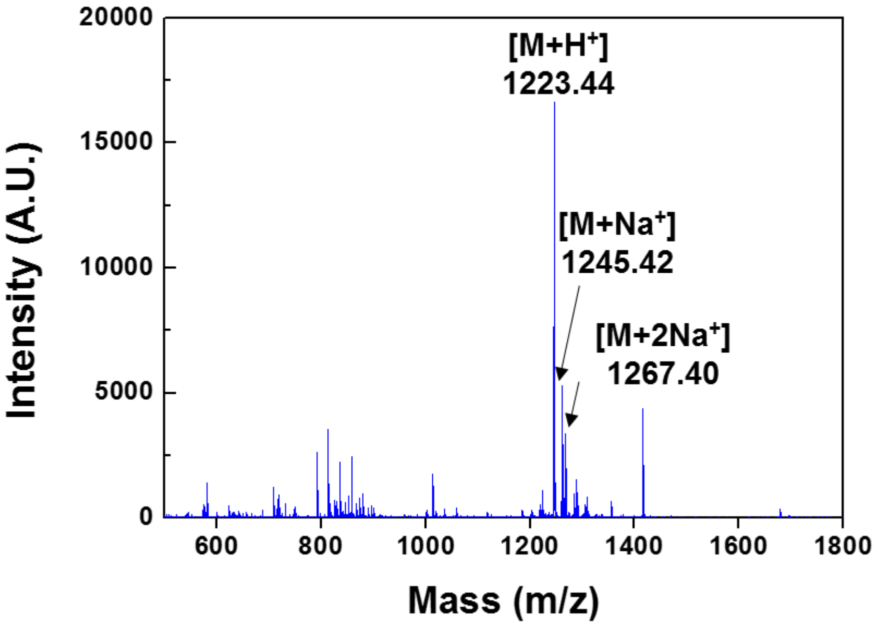

Figure S3. Molecular weight of Apo-S-Ac$_3$ManNAz was confirmed by matrix-assisted laser desorption/ionization (MALDI) analysis. The exact mass of Apo-S-Ac$_3$ManNAz was found to be 1223.44 [M+H$^+$], 1245.42 [M+Na$^+$], and 1267.40 [M+2Na$^+$] m/z.

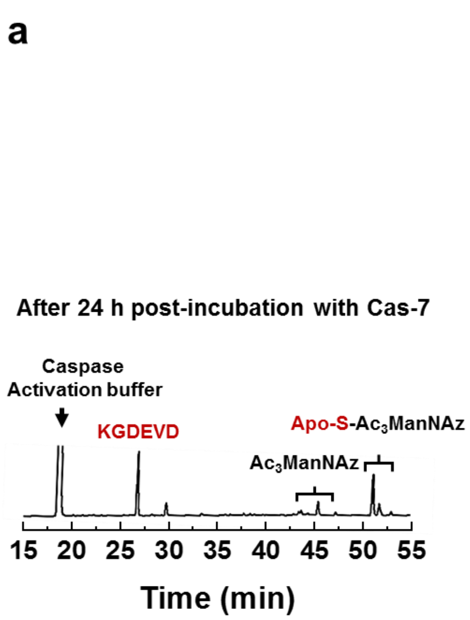

Figure S4. The *in vitro* (a) Cas-7-specific Ac3ManNAz release property of Apo-S-Ac3ManNAz was monitored by HPLC system after 24 h post-incubation with Cas-7. (b) Hydrolysis reaction of Apo-S-Ac3ManNAz was monitored by HPLC system at 0, 1, 3, 6, and 24 h.

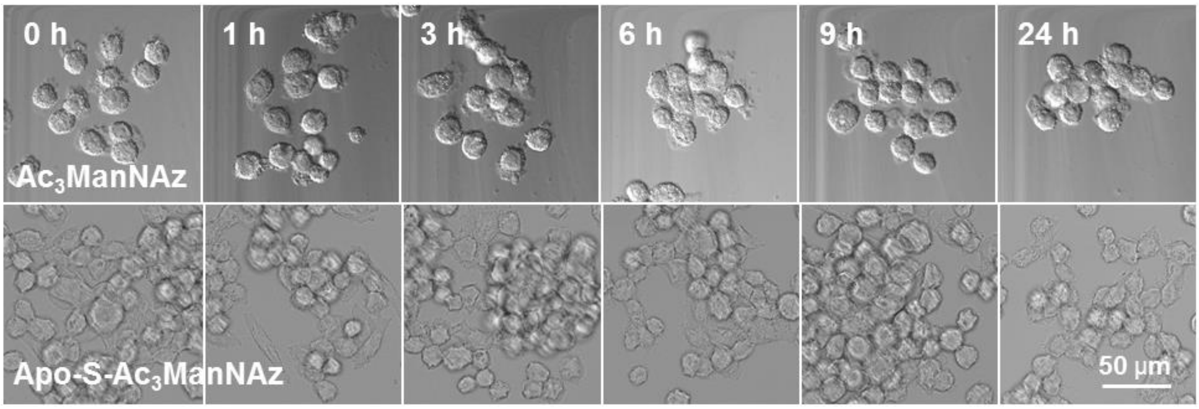

Figure S5. Differential interference contrast (DIC) images of PC-3 tumor cells incubated with Ac3ManNAz or Apo-S-Ac3ManNAz for 0, 1, 3, 6, 9, and 24 h for 37 °C (without TRAIL).

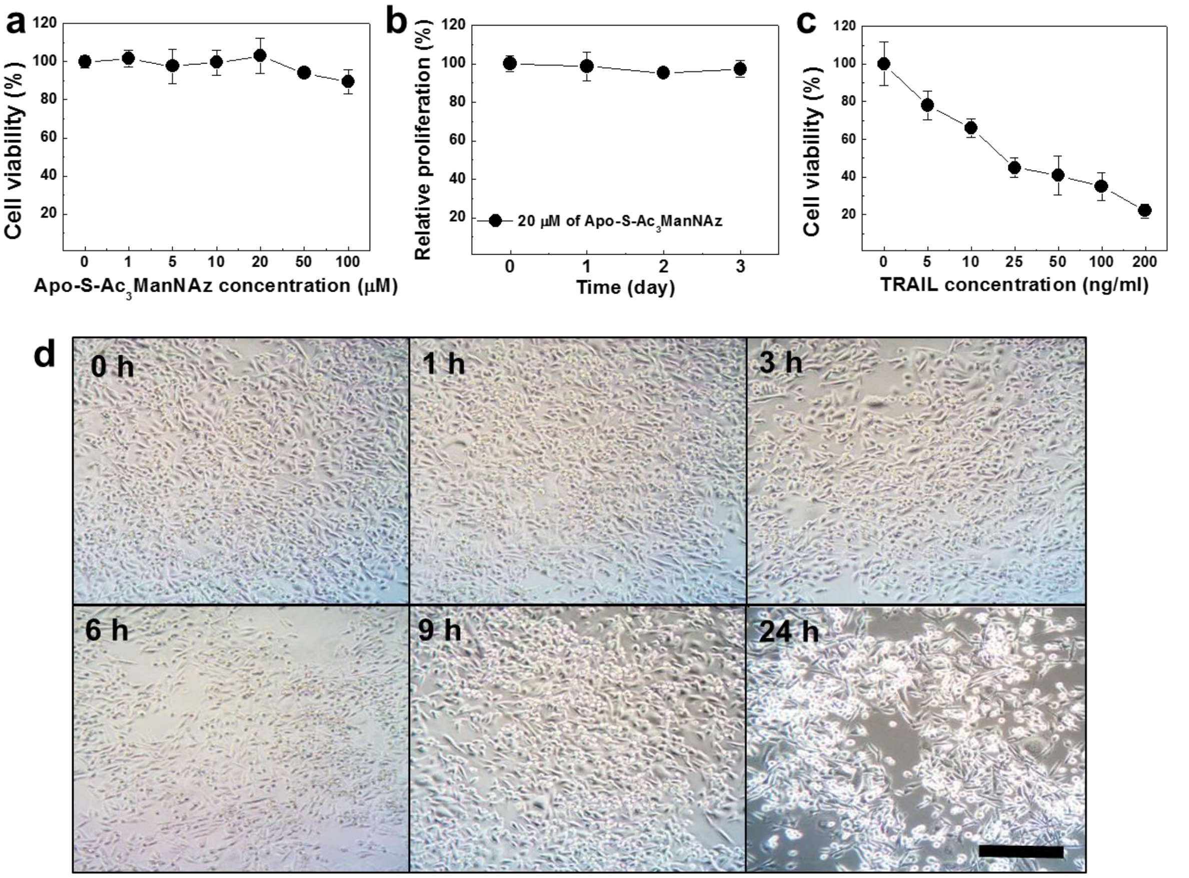

Figure S6. *In vitro* toxicity of PC-3 tumor cells. (a) Cell viability and (b) relative proliferation of Apo-S-Ac$_3$ManNAz-treated PC-3 tumor cells. (c) Cell viability of TRAIL-treated PC-3 tumor cells. (d) Optical images of PC-3 cells treated with TRAIL (7 ng) for 0, 1, 3, 6, 9, and 24 h. The scale bar indicates 200 μm.

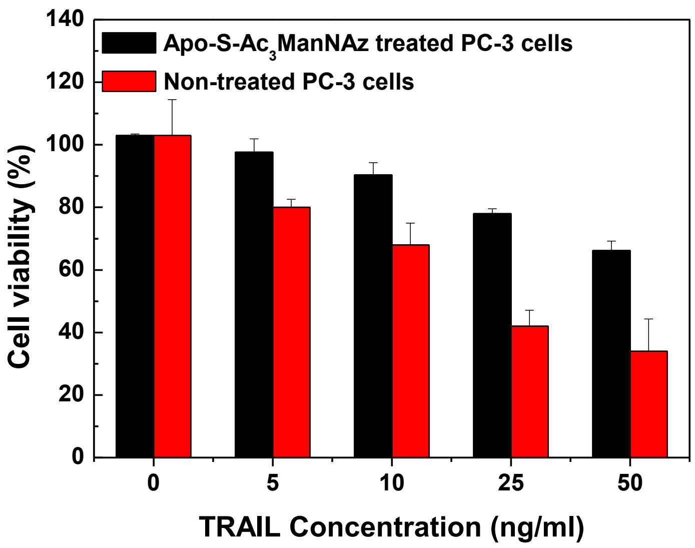

Figure S7. Cell viability of TRAIL-treated PC-3 tumor cells (24 h). As a control experiment, 20 μM of Apo-S-Ac_3ManNAz was treated to PC-3 tumor cells 24 h before TRAIL treatment to confirm the inhibition of cell death.

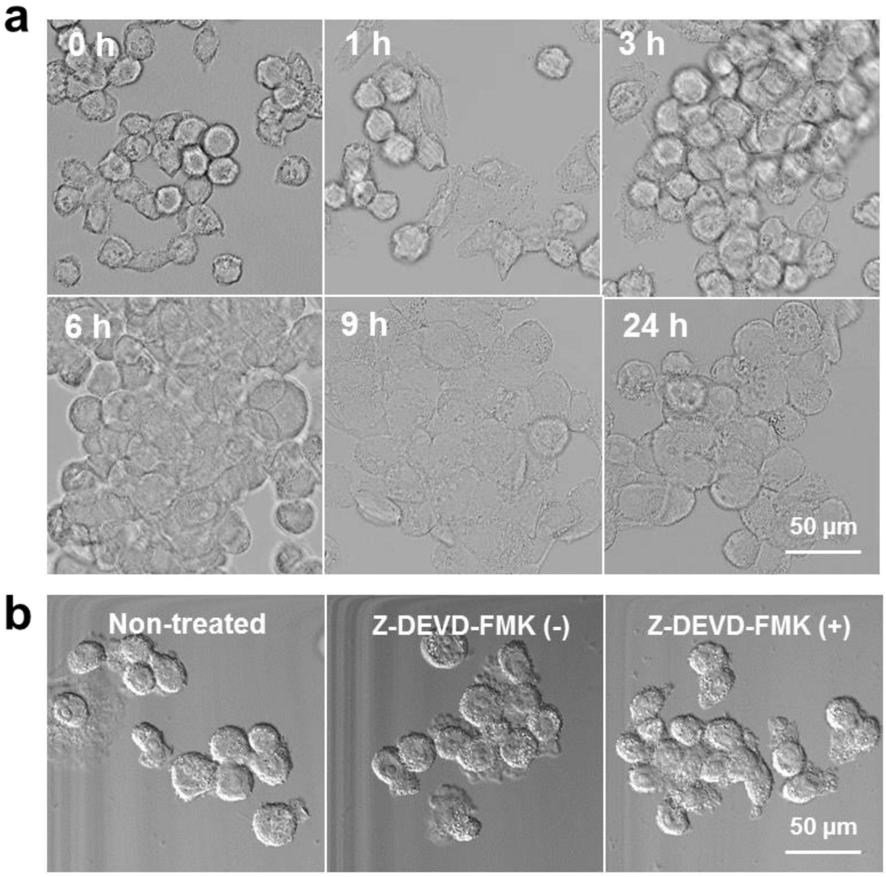

Figure S8. DIC images of (a) Apo-S-Ac_3ManNAz-treated PC-3 cells, which incubated with 7 ng/ml of TRAIL for 0, 1, 3, 6, 9, and 24 h. (b) Apo-S-Ac_3ManNAz-treated PC-3 cells, which incubated with/without z-DEVD-FMK for 24 h.

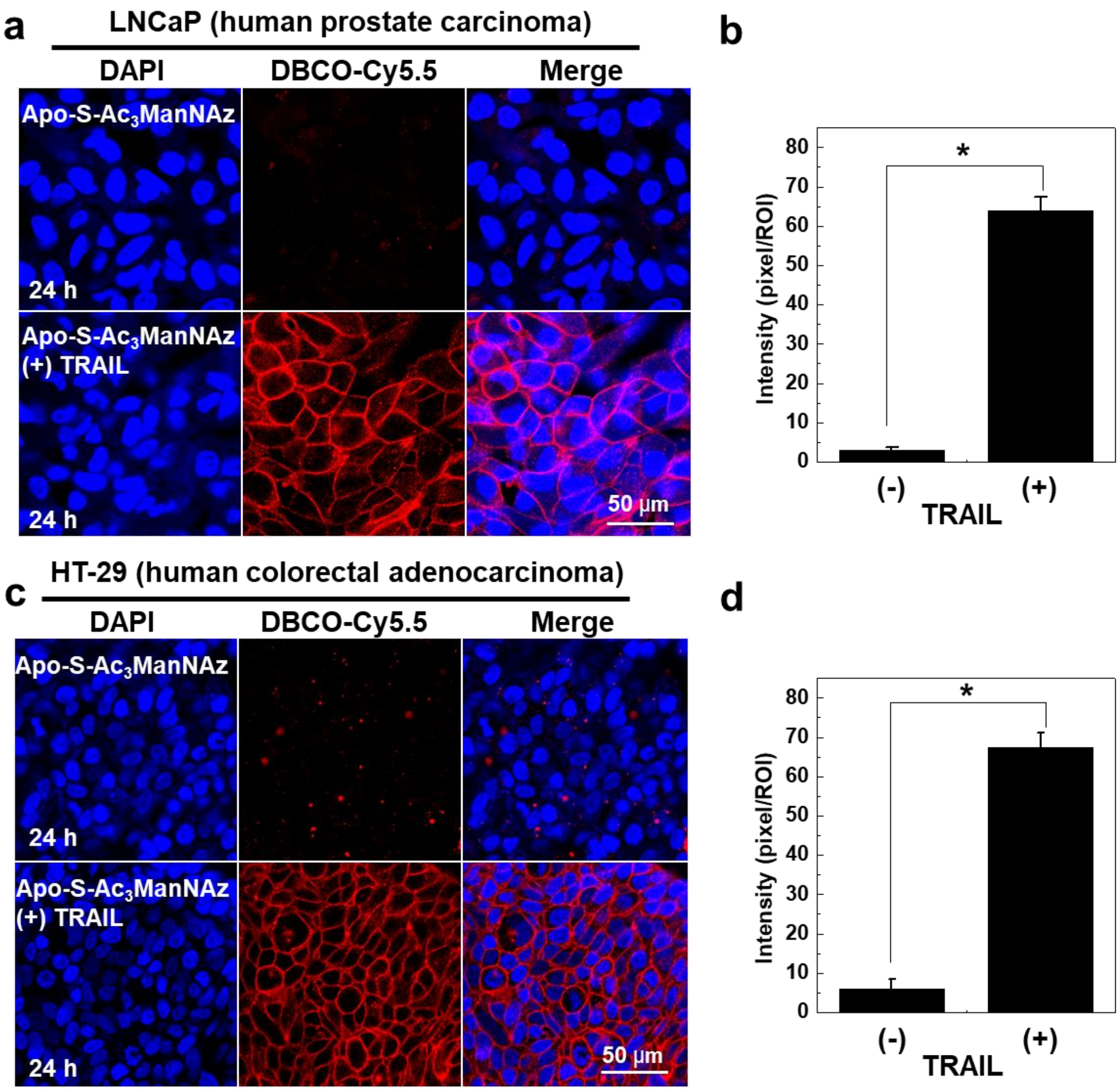

Figure S9. Confocal laser scanning microscope (CLSM) images of (a) Apo-S-Ac$_3$ManNAz (20 μM) and TRAIL (7 ng/ml)-treated LNCaP and (c) Apo-S-Ac$_3$ManNAz (20 μM) and TRAIL (7 ng/ml)-treated HT-29 tumor cells, followed by DBCO-Cy5.5 (200 nM) to visualize azido groups. Red = DBCO-Cy5.5 channel; Blue = DAPI channel. The relative intensity of (b) Apo-S-Ac$_3$ManNAz- and TRAIL-treated LNCaP and (d) Apo-S-Ac$_3$ManNAz- and TRAIL-treated HT-29 tumor cells from (a) and (c). (*) indicates difference at the $p < 0.01$ significance level.

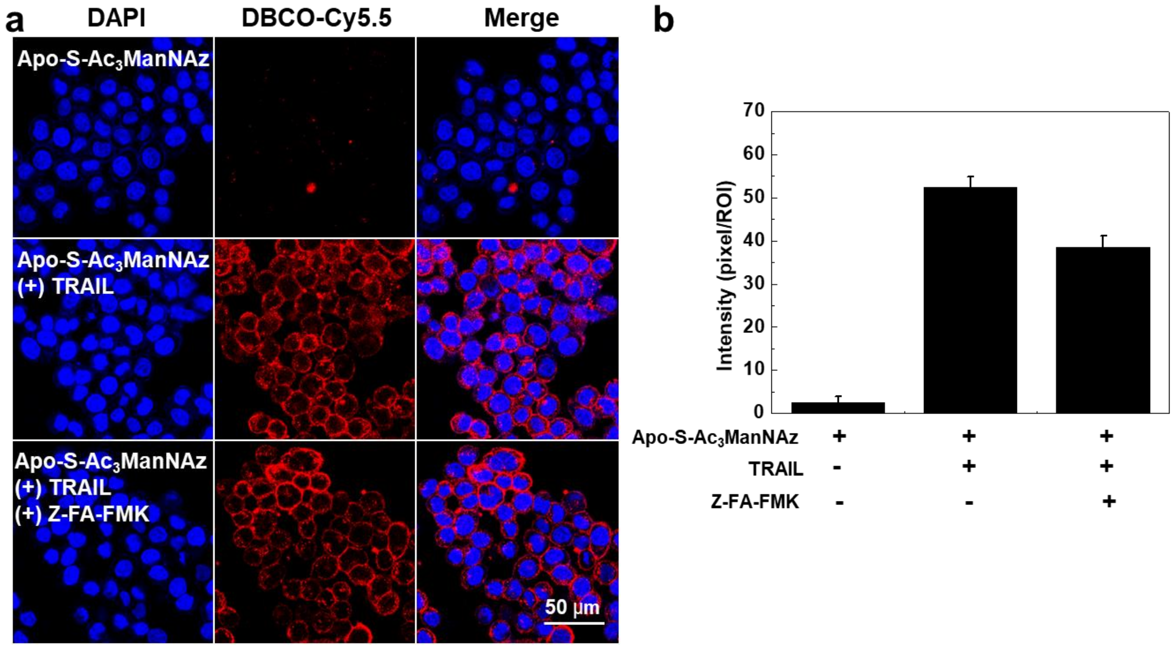

Figure S10. (a) Confocal laser scanning microscope (CLSM) images of Apo-S-Ac_3ManNAz (20 μM)-treated PC-3 tumor cells. To generate azido group on the apoptotic tumor cells, the PC-3 tumor cells were treated with TRAIL (7 ng/ml) or TRAIL/z-FA-FMK (200 μM). DBCO-Cy5.5 (200 nM) was used to visualize azido groups. Red = DBCO-Cy5.5 channel; Blue = DAPI channel. (b) The relative intensity of DBCO-Cy5.5 from (a).

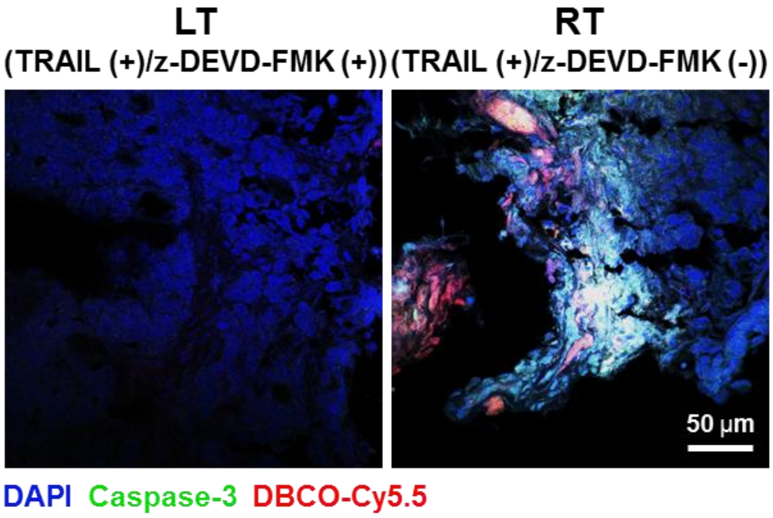

Figure S11. Merging tissue fluorescence images from **Figure 5d**. Red = DBCO-Cy5.5 channel; Blue = DAPI channel; Green = Cas-3 channel.

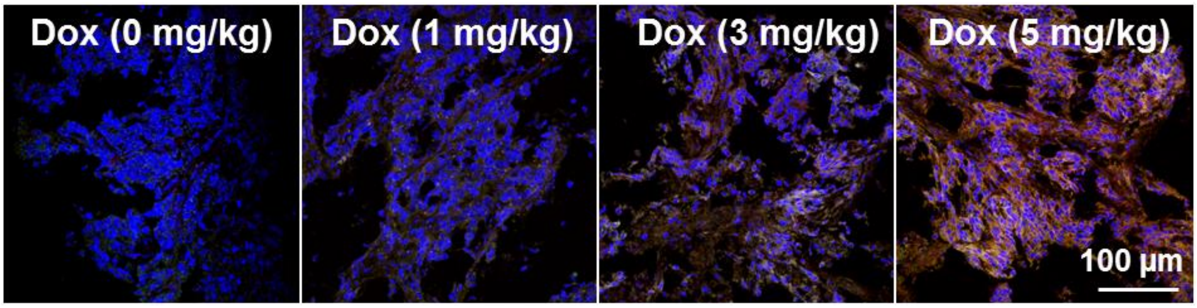

Figure S12. Tissue fluorescence merging images of PC-3 tumor tissues from **Figure 6f**.

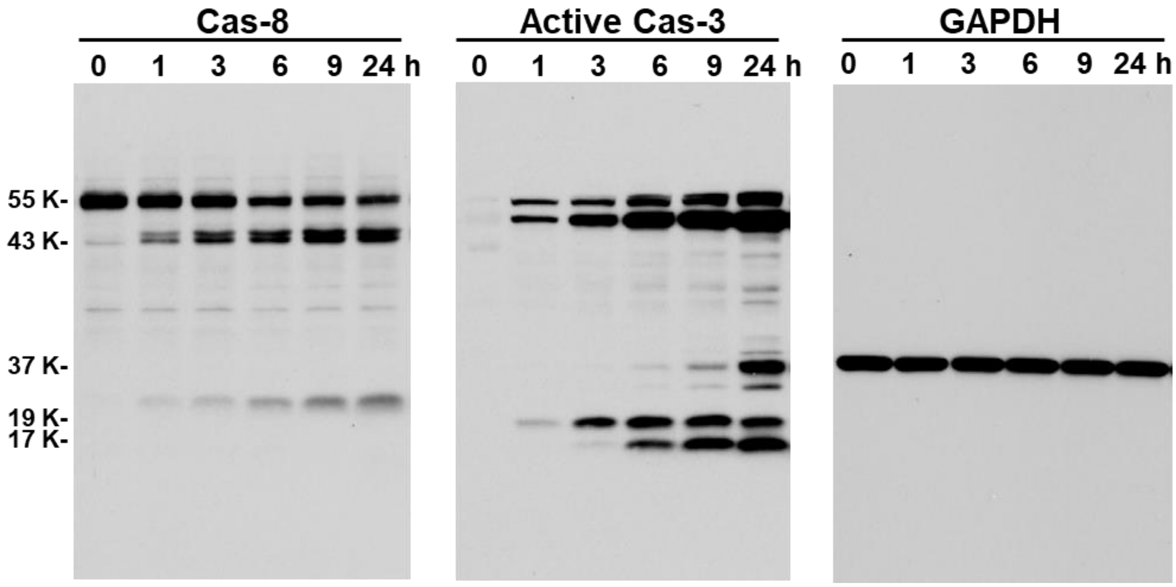

Figure S13. Full length gel images for the western blot analysis of **Figure 3a**.
